# The C-terminus of infectious bursal disease virus VP3 encodes a predicted intrinsically disordered region, which promotes the formation of cytoplasmic puncta and modulates their physical properties

**DOI:** 10.1128/mbio.03107-25

**Published:** 2026-01-12

**Authors:** A. J. Brodrick, M. Liu, G. Smith-Hicks, J. Dong, S. C. Egana-Labrin, A. J. Broadbent

**Affiliations:** 1 Department of Animal and Avian Sciences, University of Maryland 1068 , College Park, Maryland, USA; Virginia Tech, Blacksburg, Virginia, USA

**Keywords:** birnavirus, IBDV, LLPS, virus factory, phase separation, biomolecular condensate

## Abstract

The virus factories (VFs) of infectious bursal disease virus (IBDV) are biomolecular condensates formed through liquid-liquid phase separation (LLPS). A major component of the IBDV VF is the nonstructural protein VP3, but the molecular basis underlying VF formation remains poorly understood. Here, we demonstrate that VP3 was necessary but not sufficient for phase-separated biomolecular condensates to form. Using live-cell imaging of cells transfected with fluorescent reporter-tagged proteins, our data suggested that the minimal components required to form these structures were VP3, the viral polymerase (VP1), and viral RNA (vRNA). Furthermore, using protein modeling and molecular dynamics simulations, we determined that the 36 amino acid carboxy (C)-terminus of VP3 forms a highly dynamic intrinsically disordered region (IDR). When this was removed, puncta were significantly less numerous (*P* < 0.0001), smaller (*P* < 0.0001), and more irregular in shape than puncta formed in the presence of wt VP3, demonstrating that the VP3 C-terminal IDR promoted their formation. Moreover, by fluorescence recovery after photobleaching, the VP3ΔC puncta had a significantly reduced mobile fraction (0.29) as compared to full-length VP3 puncta (0.70) (*P* < 0.001), demonstrating that the VP3 C-terminal IDR modulated their physical properties. In summary, our data reveal that VP3 forms part of a higher-order complex with VP1, and likely vRNA, to drive LLPS and the formation of IBDV VFs, and that the VP3 C-terminus encodes an IDR that is essential for modulating the physical properties of the resultant structures.

**IMPORTANCE:**

Liquid-liquid phase separation (LLPS) is a phenomenon of growing interest in cell biology. It is a part of the replication cycles of diverse viruses, but our understanding of the molecular basis that underpins the mechanism of phase separation is incomplete. We previously demonstrated that the virus factories of the birnavirus IBDV, a major agricultural pathogen, are biomolecular condensates formed through LLPS. In this study, we discovered that VP3 was necessary but not sufficient for condensates to form, and the minimal components of these structures were VP3, VP1, and likely vRNA. We also discovered that the C-terminal 36 amino acid region of IBDV VP3 encoded a highly dynamic intrinsically disordered region that promoted the formation of the cytoplasmic puncta and modulated their physical properties. This work contributes to a more detailed understanding of birnavirus replication at the molecular level and to the study of LLPS as a phenomenon.

## INTRODUCTION

Viruses must regulate and organize biochemical processes to replicate efficiently, yet they also face strong selective pressures for gene and protein economy. One mechanism that achieves both of these goals is liquid-liquid phase separation (LLPS), wherein biomolecules spontaneously form liquid condensates in a liquid medium, separated from the surroundings by a phase transition barrier rather than the lipid membranes of typical organelles (1). LLPS is exploited by numerous viruses in the formation of their replicative compartments, including SARS-CoV-2 (2), adenoviruses (3), rabies viruses (4), Ebola viruses (5), measles virus (6), influenza viruses (7), and viruses with double-stranded RNA (dsRNA) genomes including reovirus (ReoV) (8), rotavirus (9), and birnaviruses (10).

LLPS is driven by the formation of molecular assemblages from biomolecules capable of multivalent interactions. It is distinguished from aggregation by the characteristically weak strength of the interactions, which permit diffusion of the biomolecules, while the cumulative effect of the interactions maintains the energetic favorability of phase separation (11, 12). Constituent components of biomolecular condensates formed through LLPS can be broadly categorized as “scaffold” or “client” biomolecules (13). Scaffold biomolecules are essential for phase separation to occur and contribute to the requisite multivalency that drives the phenomenon, whereas client biomolecules are recruited and phase separate, but would not do so in the absence of the scaffolds (13). The requirement of multivalent and weak interactions for phase separation means that intrinsically disordered proteins (IDPs) and proteins with intrinsically disordered regions (IDRs) are commonly found in LLPS structures, as these proteins and domains lack a single well-defined secondary structure, enabling them to participate in numerous weak or transient interactions as they explore their conformation space (12, 14). However, the requisite multivalency for phase separation can also be achieved through structured protein domains, meaning IDPs or IDRs are not absolutely required for LLPS.

In ReoV, the non-structural protein µNS is considered to be a scaffold protein, which self-assembles into phase-separated structures in the absence of other viral components (15), yet only the extreme N- and C-terminal sequences of µNS are likely to be disordered, and within the minimal region responsible for the formation of viral factory-like structures, there are two predicted alpha helices that have been proposed as principal interacting elements for µNS self-assembly (15). By contrast, in rotavirus, both NSP2 and NSP5 are required to drive LLPS. NSP5 is an IDP that can assemble into several higher-ordered oligomers (16) and the carboxy (C)-terminal region (CTR) of NSP2 is flexible, allowing it to participate in domain-swapping interactions that may be important in viroplasm formation (17). Despite these advances in our understanding of the molecular basis of phase separation in dsRNA viruses such as reoviruses and rotaviruses, comparatively little is understood regarding the mechanism underpinning the phase separation of the replicative compartments formed by birnaviruses.

Birnaviruses are non-enveloped, dsRNA viruses with a bipartite genome. Members of the *Birnaviridae* family include infectious bursal disease virus (IBDV) and infectious pancreatic necrosis virus, which are pathogens of birds and fish, respectively, and are of economic importance to the poultry and aquaculture industries. IBDV encodes five viral proteins (VP1–5) across two genome segments, with segment A encoding a polyprotein that is cleaved to yield VP2, 4, and 3 and segment B encoding the polymerase, VP1. Previously, we discovered that the viral replicative compartments of the *Birnaviridae* family, termed “virus factories” (VFs), also form through LLPS (10), but the molecular basis for the phase separation of IBDV VFs has yet to be identified. A major component of the IBDV VF is viral protein 3 (VP3) (10, 18). In this study, we employed computational, cell culture, and biochemical approaches to better characterize the role IBDV VP3 plays in LLPS. We discovered that VP3 was necessary but not sufficient for cytoplasmic puncta to form and that the minimal components required for the formation of phase-separated biomolecular condensates were VP3, VP1, and likely viral RNA (vRNA). Moreover, we identified a predicted 36-residue C-terminal IDR in VP3 that we demonstrate promotes the formation of cytoplasmic puncta and modulates their physical properties, thus contributing to our knowledge of this virus and to the field of LLPS.

## RESULTS

### VP3 is necessary but not sufficient to drive the formation of cytoplasmic puncta

**Fig 1:**
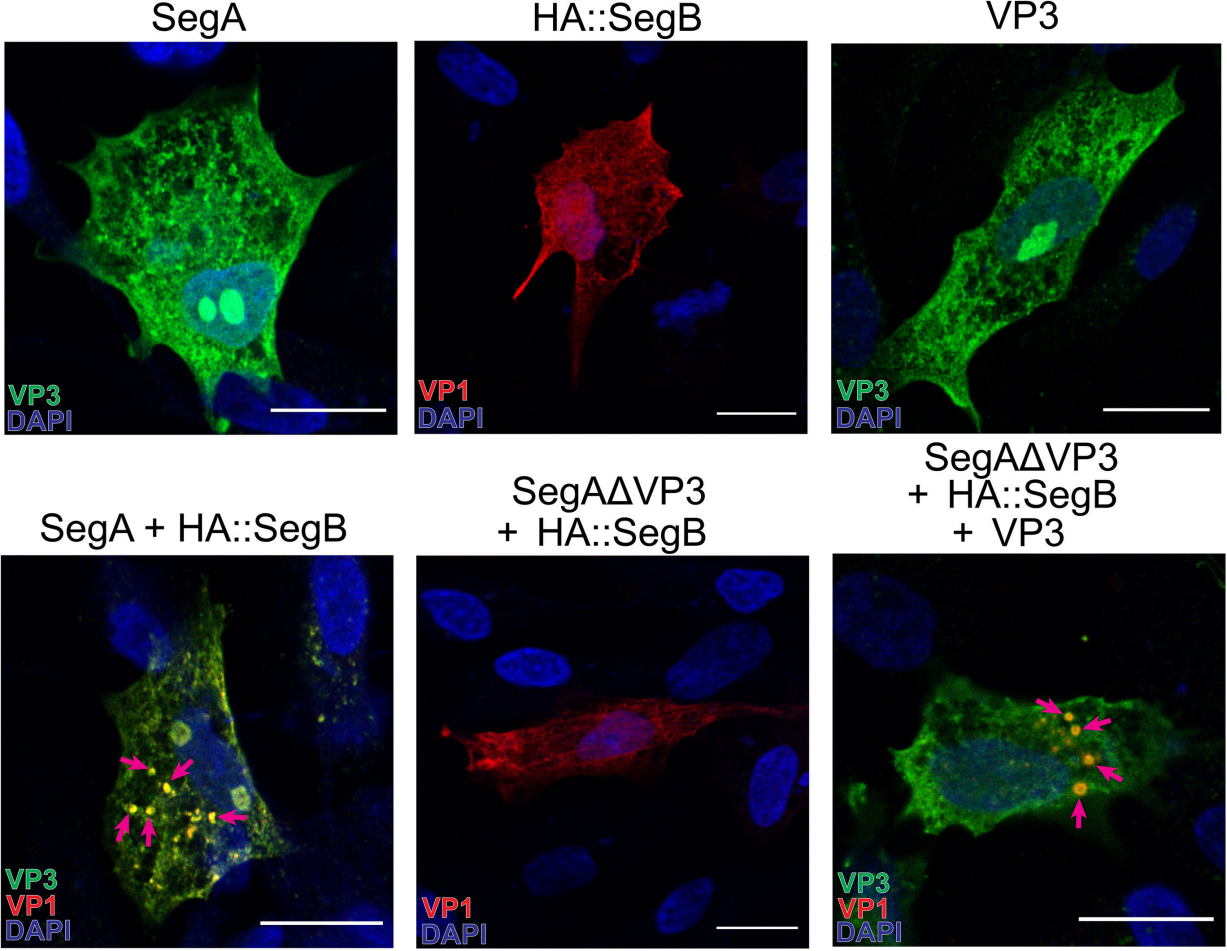
VP3 is necessary, but not sufficient, for the formation of IBDV cytoplasmic puncta. DF-1 cells were transfected with Segment A (SegA), HA::Segment B (HA::SegB), VP3 alone, or SegA + HA::SegB, SegAΔVP3 + HA::SegB, or SegAΔVP3 + HA::SegB + VP3 (**A**). Cells were fixed at 34 hpt and stained with DAPI (blue) and antibodies against VP3 (green) or HA (red). Mosaic images were acquired with the Leica Stellaris 8 scanning confocal microscope. Arrows denote puncta; scale bars, 50 µm.

Previously, we determined that co-transfecting cells with reverse genetics plasmids encoding segments A and B results in the rescue of infectious IBDV (19). To determine the necessity of VP3 in the formation of LLPS structures, here we generated a truncated variant of segment A lacking the VP3 coding sequence (CDS) (SegAΔVP3). When DF-1 cells were transfected individually with wild-type (wt) segment A (SegA) or a wt segment B containing an HA tag fused to the N-terminus (HA::SegB), VF-like punctate structures were not observed, but when cells were co-transfected with SegA and HA::SegB together, VF-like puncta were observed, as expected. In contrast, VF-like puncta were not observed in cells that were co-transfected with SegAΔVP3 and HA::SegB together, although it was not possible to image SegAΔVP3 directly, as the antibody we used was raised against VP3. Furthermore, when cells were co-transfected with SegAΔVP3, HA::SegB, and a wt VP3 under the control of a CMV promoter (VP3), the punctate phenotype was recovered (Fig. 1). Taken together, these data indicated that IBDV VP3 was necessary but not sufficient for the formation of puncta.

**Fig 2:**
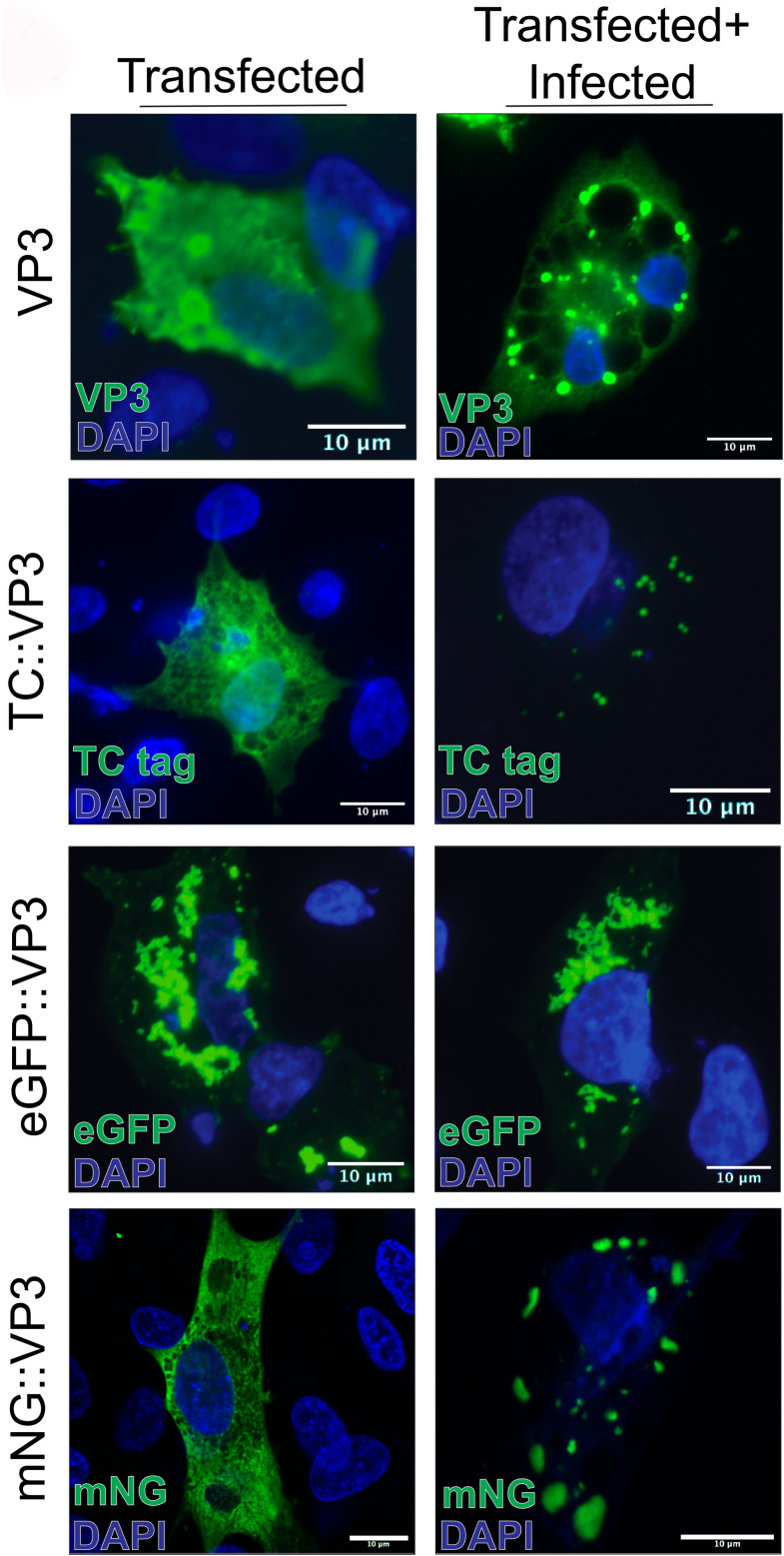
Evaluating the expression of VP3 fused to different tags. DF-1 cells were transfected with a vector expressing VP3, or reporter-conjugated VP3 constructs (TC::VP3, eGFP::VP3, and mNG::VP3). Cells were imaged by confocal microscopy at 18 hpt, either alone (left column) or in cells simultaneously transfected with reporter::VP3 conjugates and infected with strain PBG98 IBDV (multiplicity of infection [MOI] = 5). Scale bars, 10 µm.

These data were at odds with our previous report that eGFP::VP3 expressed alone formed cytoplasmic puncta (10). We therefore surmised that the eGFP tag influenced the distribution of VP3 in the cell. As it was necessary to generate VP3 fusion proteins to conduct live-cell imaging experiments, we therefore prepared a panel of VP3 proteins fused to different reporters to select one that better represented the true distribution of VP3 in the cytoplasm. All tags were fused to the N-terminus of VP3, as previous reports revealed that C-terminal fusions prevented the rescue of recombinant viruses by reverse genetics and might therefore interrupt the function(s) of the VP3 C-terminus (20). Reporters included tetracysteine (TC), enhanced green fluorescence protein (eGFP), and mNeonGreen (mNG) (21) (Fig. 2). Cells transfected with TC::VP3 were chemically labeled with FLaSH prior to imaging, and cells transfected with an untagged VP3 control were analyzed by immunofluorescence microscopy with a mouse monoclonal antibody raised against full-length VP3 (α-VP3) (22). When expressed alone, untagged VP3 assumed a predominantly diffuse or moderately reticulated cytoplasmic staining pattern in transfected DF-1 cells, and the pattern of the TC::VP3 and mNG::VP3 fluorescence signals was also consistent with this observation. In contrast, eGFP::VP3 formed foci similar to our previous observations. Based on these data, we conclude that the eGFP::VP3 foci did not represent the true distribution of untagged VP3 in the cell, and so subsequent experiments were not performed with this reporter. When we simultaneously co-transfected DF-1 cells with the reporter::VP3 plasmids and infected them with IBDV strain PBG98, we observed a redistribution of the fluorescence signal into cytoplasmic puncta. These data demonstrate that when expressed alone, VP3 did not spontaneously phase separate into cytoplasmic puncta; however, when expressed in infected cells, VP3 was recruited to the VFs. We selected mNG as a reporter for use in subsequent experiments due to its nature as a constitutively fluorescent protein, functionality in live cells, known low propensity for multimerization (15, 21), and performance in our screen.

### VP3 and VP1, in the presence of a vRNA template, are the minimal components necessary for the formation of phase-separated biomolecular condensates

**Fig 3:**
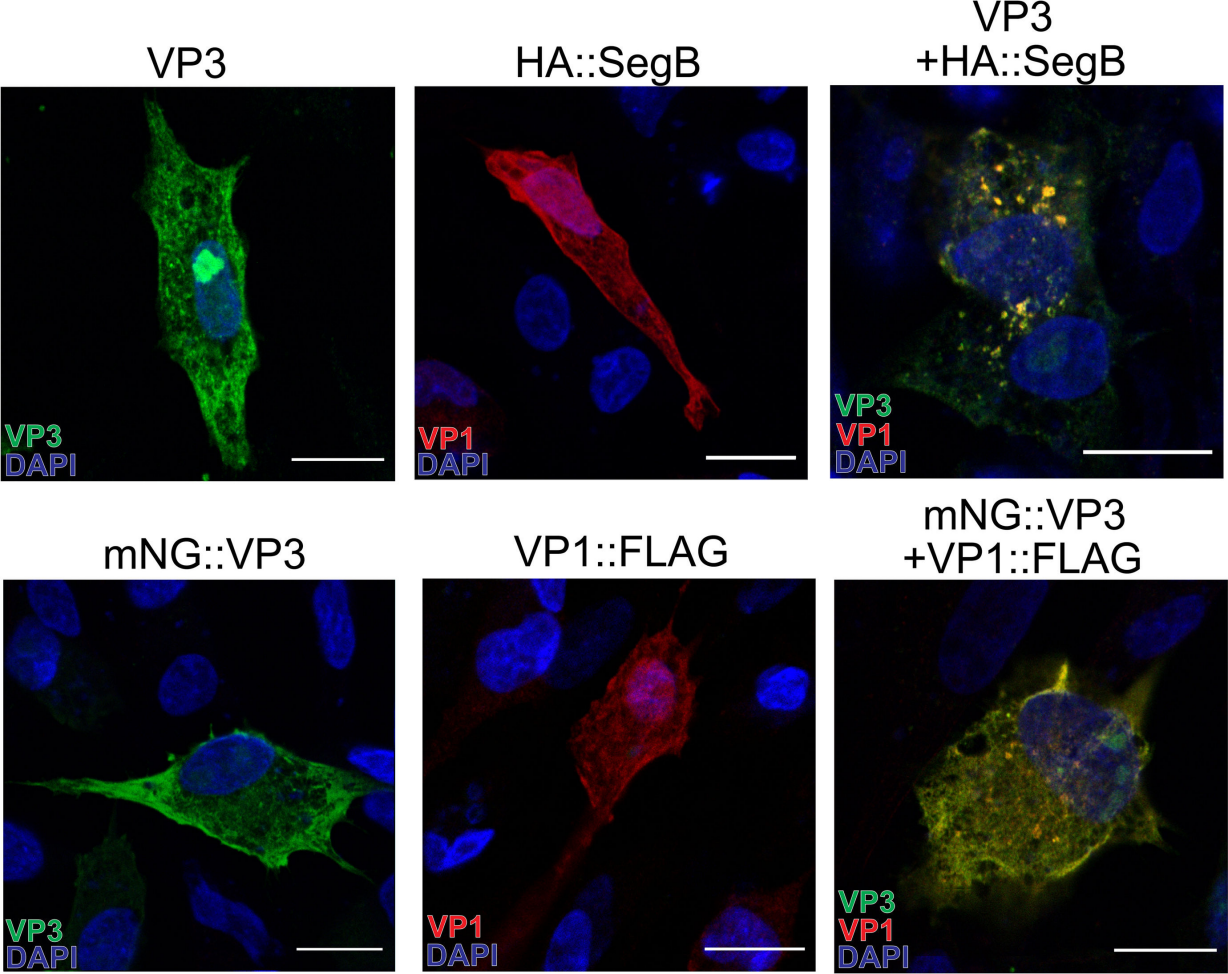
The minimal components of IBDV cytoplasmic puncta are VP3 and VP1. DF-1 cells were transfected with mNG::VP3, VP1::FLAG, VP3, HA::Segment B (HA::SegB) or a mixture of VP3 + HA::SegB (**A**). Cells were fixed at 34 hpt, and the nuclei were stained with DAPI (blue). mNG is shown in green, Segment B (visualized by staining with anti-HA monoclonal antibody) and VP1 (visualized by staining with anti-FLAG monoclonal antibody) are shown in red. Mosaic images were acquired with the Leica Stellaris 8 scanning confocal microscope. Scale bars, 50 µm.

**Fig 4:**
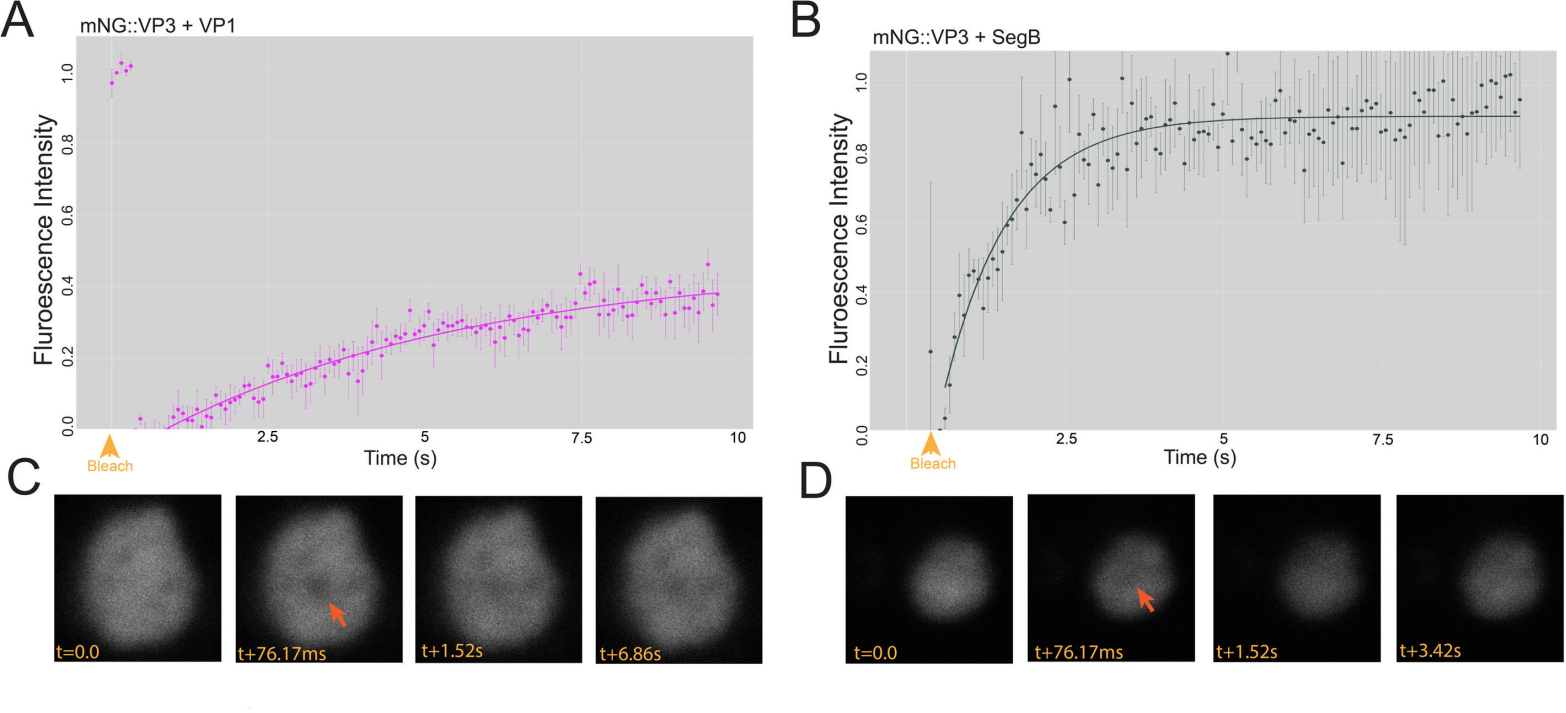
The minimal components of IBDV phase-separated biomolecular condensates are VP3, VP1, and RNA. DF-1 cells were co-transfected with a mixture of mNG::VP3 and VP1::FLAG, and a time-lapse series was captured by live confocal microscopy at 34 hpt with a single iteration of point bleaching on the 5th frame (yellow arrowhead). Intensity data were extracted and used to calculate a FRAP recovery curve. Results are from three replicates; error bars = SEM (**A**). DF-1 cells were co-transfected with a mixture of mNG::VP3 and SegB, and a FRAP recovery curve was generated in the same way (**B**). Representative recovery images for a photobleached puncta formed in the presence of VP1::FLAG with the time post-bleach (t) shown in milliseconds (ms) (**C**). Representative recovery images for a photobleached puncta formed in the presence of SegB with the t post-bleach shown in s (**D**).

Next, we sought to determine the minimal components of IBDV phase-separated biomolecular condensates. As SegA and SegB form the reverse genetics system for IBDV, they express both mRNA and non-capped RNA that the VP1 polymerase can use to synthesize the ds vRNA. As SegA did not lead to the formation of cytoplasmic puncta and encodes VP2, 3, 4, and 5, we concluded that VP3 did not form condensates in the presence of these other proteins. As SegB did not lead to the formation of cytoplasmic puncta and encodes VP1, which synthesizes vRNA, we concluded that VP1 and vRNA did not lead to the formation of condensates in the absence of other factors. As co-transfection of SegA and SegB did form cytoplasmic puncta, we hypothesized that VP3 and VP1, either in the presence or absence of vRNA, would be necessary and sufficient for the formation of biomolecular condensates. To test this hypothesis, DF-1 cells were transfected with plasmids encoding VP3, VP1 fused to FLAG at the C-terminus (VP1::FLAG), or HA::SegB. No VF-like cytoplasmic puncta were observed in cells expressing VP3, VP1::FLAG, or HA::SegB alone. In contrast, cytoplasmic puncta were observed in cells co-expressing VP3 and HA::SegB, demonstrating that puncta were formed in the presence of VP3, VP1, and vRNA (Fig. 3). It was not possible to image cells co-expressing VP3 and VP1::FLAG as the antibodies against VP3 and FLAG were the same isotype. Therefore, we imaged cells co-expressing mNG::VP3 and VP1::FLAG and also observed puncta (Fig. 3), demonstrating that the minimal components required for cytoplasmic puncta to form were VP3 and VP1. However, as the presence of cytoplasmic puncta does not necessarily equate to phase-separated biomolecular condensates, we next imaged cells co-expressing mNG::VP3 and VP1 under the control of a CMV promoter, or mNG::VP3 and SegB by live-cell confocal microscopy. At 34 h post-transfection, the puncta in cells expressing mNG::VP3 and VP1 showed limited recovery by fluorescence recovery after photobleaching (FRAP), with a mobile fraction of 0.465. In contrast, the puncta in cells expressing mNG::VP3 and SegB showed much more robust FRAP recovery with a mobile fraction of 0.911, indicative of true liquid-like characteristics (Fig. 4). As the plasmid encoding VP1 under the control of a CMV promoter only expresses the VP1 protein, whereas the plasmid encoding SegB both encodes VP1 and provides a template for VP1-driven vRNA synthesis, these data demonstrated that the minimal components required for IBDV phase-separated biomolecular condensates to form were VP and VP1, and may include vRNA.

### VP3 encodes a C-terminal domain with a predicted lack of secondary structure

**Fig 5:**
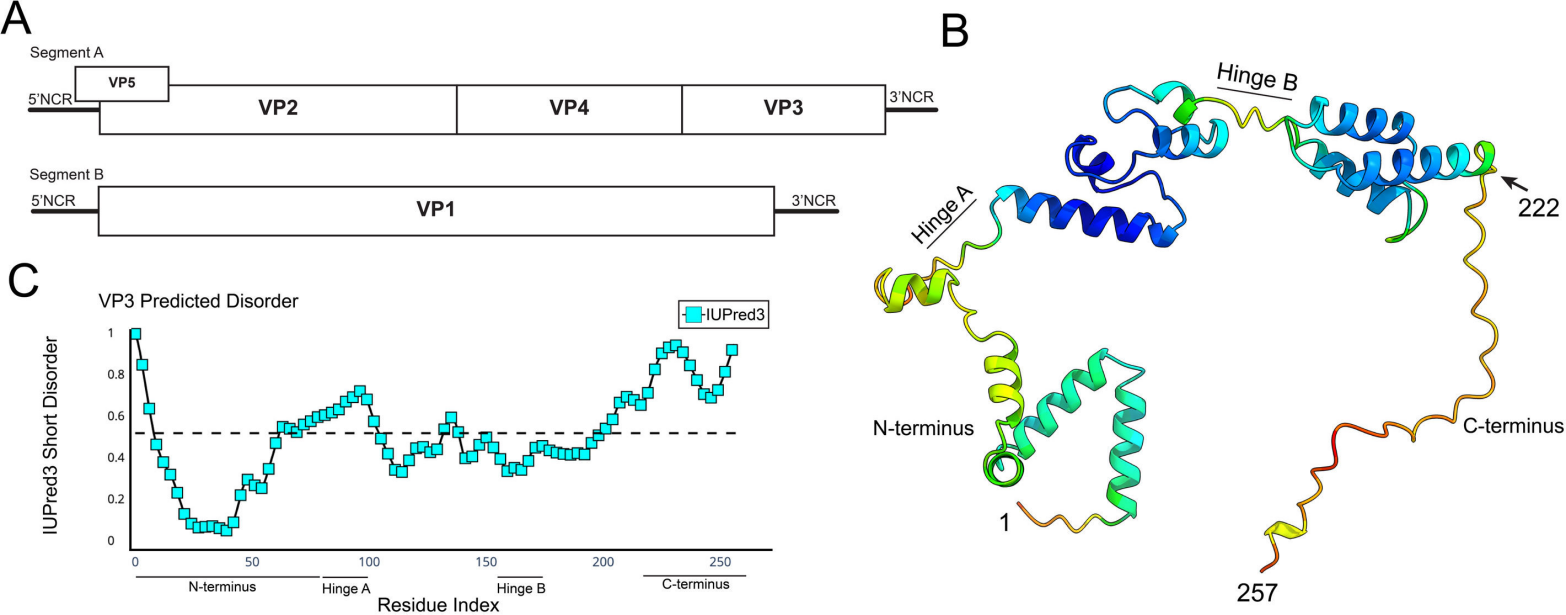
VP3 encodes a C-terminal domain with a predicted lack of secondary structure. A schematic of the IBDV genome architecture depicting Segment A encoding VP3 at the 3′ end of the coding strand, upstream of the 3′ non-coding region (NCR) and downstream of VP4 (**A**). A predicted structure for VP3, generated by AlphaFold2, visualized with UCSF ChimeraX, and colored by positional confidence (red indicates lower confidence; blue indicates higher confidence). The N-terminus, C-terminus, and hinge regions are labeled (**B**). A disorder plot of the VP3 from IBDV strain PBG98, with IUPred3 short disorder plotted on the *y*-axis against VP3 amino acid residue number on the *x*-axis. The horizontal dashed line represents an IUPred3 Short Disorder of 0.5 and the N-terminus, C-terminus, and hinge regions of VP3 are labeled (**C**).

Given that VP3 is a major component of the IBDV VF structures (23), we hypothesized that VP3 would act as a scaffold protein and show structural features conducive to driving LLPS. Only the structure of the VP3 central domains (residues 82–220) and amino terminal domain has been solved (24, 25), and so we used AlphaFold2 to model the structure of the full-length IBDV VP3 monomer from IBDV strain PBG98 (Fig. 5B). The prediction was in good agreement with the crystal structures, but notably lacked secondary structure in the C-terminal 36 residues (residues 221–257), hereafter referred to as the C-terminus. This correlated with a large predicted aligned error (PAE) in residue pairs in the C-terminus (Fig. S1A), suggesting a low confidence in the position of these residues relative to the rest of the structure (26). Moreover, these data were in agreement with the results of a disorder prediction algorithm (IUPred3 Short Disorder [27]), which indicated an increased propensity for disorder in the C-terminus as well as the N-terminus, and within two predicted “hinge” regions between structured portions of the central domains (Fig. 5C). This result inversely correlated with the Shannon entropy of the sequence (28) (Fig. S1B). Taken together, the residues of the C-terminus lacked predicted secondary structure, showed high IUPred3 disorder scores, and reduced sequence entropy, which are typical of a low-complexity IDR when measured by classical methods (29).

### The VP3 C-terminal domain is predicted to be a highly dynamic IDR

**Fig 6:**
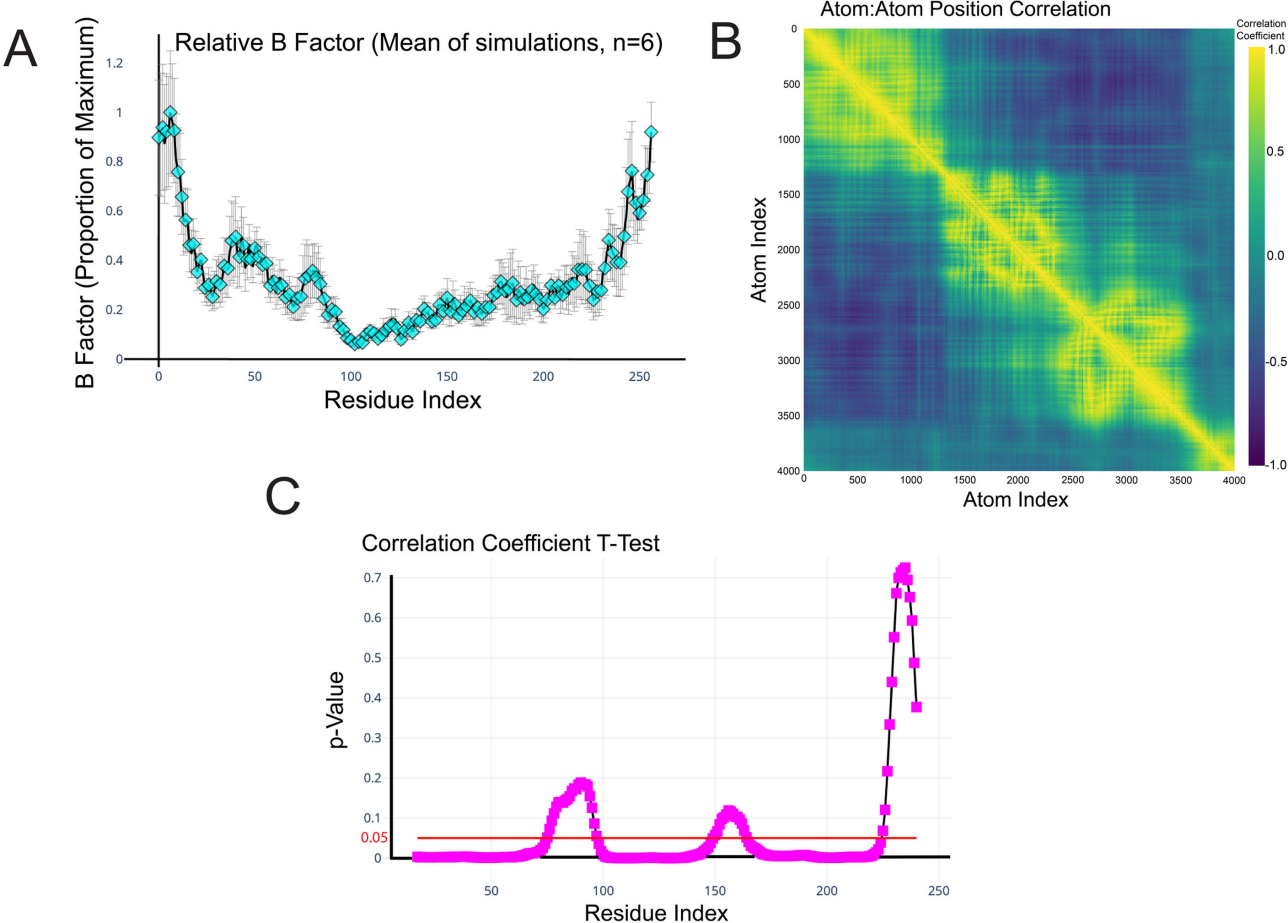
The VP3 C-terminal domain is predicted to be a highly dynamic IDR. The mean relative B-factor, calculated from MD simulations of the VP3 monomer (6 independent 20 nanosecond simulations), was plotted on the *y*-axis against VP3 amino acid residue number on the *x*-axis. Error bars represent the standard error of the mean (SEM) (**A**). Pearson’s product moment correlation coefficient between the trajectories of each pair of atoms was calculated and plotted as a heatmap. Yellow represents perfect positive correlation, purple represents perfect negative correlation, and blue/teal suggests a lack of correlation (**B**). The movement of the atoms (average positional correlation coefficient) within the 36 amino acids of the C-terminus was compared to equally sized windows of 36 amino acids in length spanning the whole length of the VP3 protein sequence, and the *P*-values were plotted on the *y*-axis against the VP3 amino acid residue number on the *x*-axis. Regions of the VP3 region that were significantly more ordered than the C-terminus had a *P* value below the 0.05 cutoff (red line) (**C**).

Molecular dynamics (MD) simulations of the VP3 monomer were employed to explore the predicted behavior of the C-terminus compared to VP3 as a whole. Each simulation was 20 nanoseconds in duration, and the alpha carbons of VP3 were visualized as animated three-dimensional scatter plots of atomic coordinates (Movie S1). These data demonstrated that the C-terminus explored a greater breadth of the conformation space than other domains of the VP3 monomer. To quantify the relative mobility of each residue, six independent simulations were run, and B factor, a metric associated with thermal motion, was calculated for each alpha carbon in each simulation. The average per-atom B factor values were plotted, which revealed an elevated B factor in the C-terminus (Fig. 6A), demonstrating its increased predicted mobility. However, the B factor is an imperfect metric for distinguishing order from disorder, as ordered regions of proteins can undergo substantial thermal motion in MD simulations if they are adjacent to flexible “hinge” or “loop” regions (30). Therefore, to determine whether the behavior of the C-terminus differed from other regions of the protein, we calculated pairwise correlation coefficients for the movement of atoms across the VP3 monomer (atomic motion correlation analysis) and plotted the results as a heatmap (Fig. 6B). This revealed that the movement of the C-terminus was not well correlated with other regions of the protein (Fig. 6B, bottom right corner), suggesting it moves independently of the rest of the protein. As the C-terminus is 36 amino acids in length, we next determined the atomic motion of equally sized sections of 36 amino acids in length across the whole VP3 protein and compared these data to the C-terminus, to determine whether a given region was significantly more ordered than the C-terminus or not (Fig. 6C). If a given region of the protein was significantly more ordered than the C-terminus, it had a *P* value below the 0.05 cutoff (red line), whereas if a particular region was not significantly more ordered than the C-terminus, then the *P* value was higher and hence gave a peak (Fig. 6C). The VP3 protein was significantly more ordered than the C-terminus at all regions except the C-terminus itself, and amino acids 74–96 and 149–163, both of which correspond to the predicted unstructured hinge regions of the protein, and the latter of which corresponds to a “loop” region in the VP3 central domain structure solved by X-Ray diffraction (24). Taken together with the calculated B-factor values and the broad conformational landscape explored by the C-terminus, the results of MD simulations demonstrated that the VP3 C-terminus is predicted to be a highly dynamic IDR. Moreover, when we performed MD simulations on a panel of VP3s from four diverse IBDV strains from different genogroups, all exhibited the same elevated B factor in the C-terminus, and there was no statistically significant difference between them (Fig. S2), demonstrating that the VP3 C-terminal IDR is present in multiple IBDV strains.

### IBDV VP3 can exist as a homodimer with two C-terminal dynamic IDRs extending from the core

**Fig 7:**
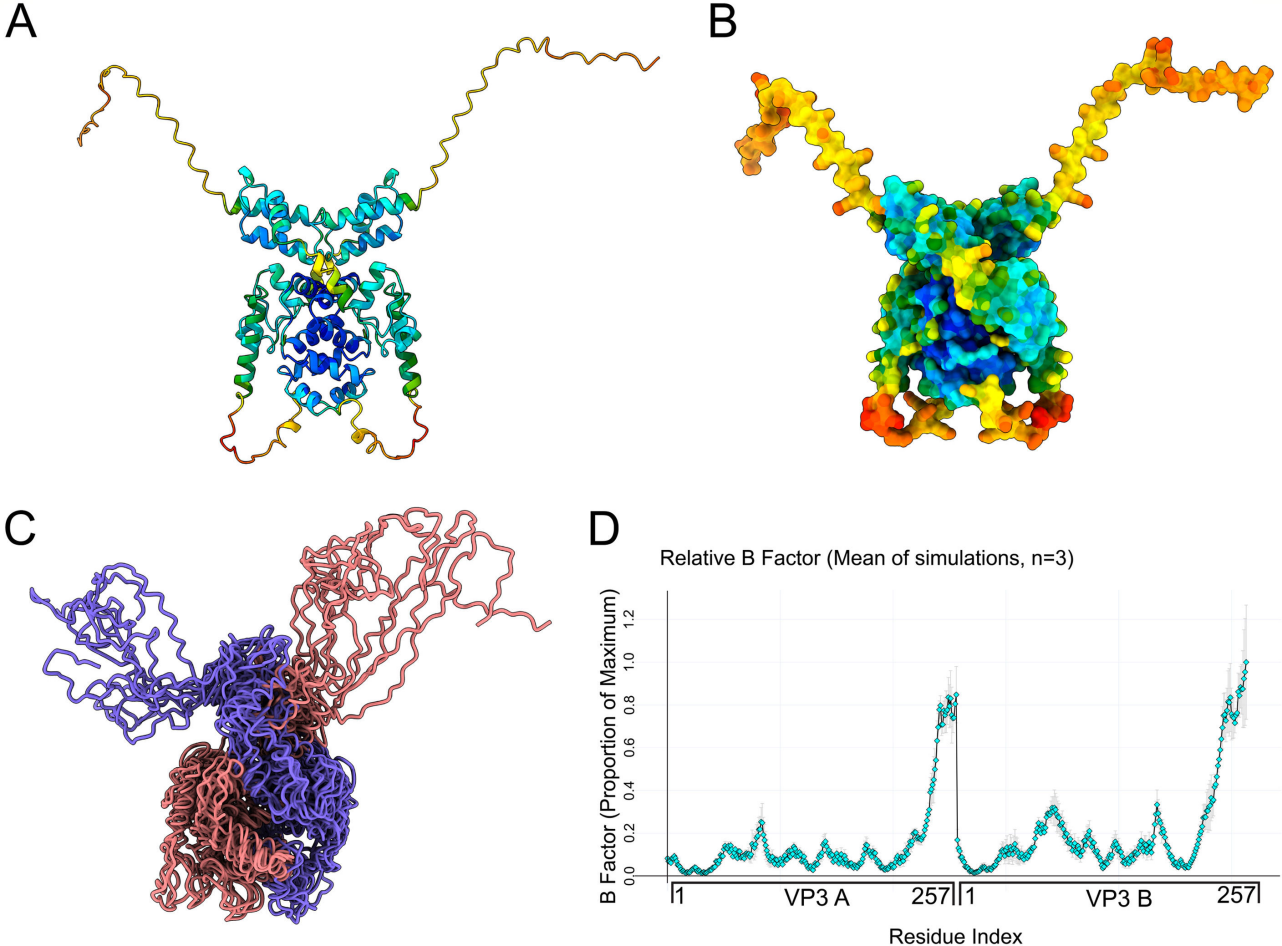
IBDV VP3 can exist as a homodimer with two C-terminal dynamic IDRs extending from the core. A cartoon rendering of the VP3 homodimer as predicted by AlphaFold3, visualized in UCSF ChimeraX, and colored by positional confidence (red indicates lower confidence; blue indicates higher confidence) (**A**). A solvent-accessible surface of the VP3 homodimer, visualized by UCSF ChimeraX, and colored by positional confidence (red indicates lower confidence; blue indicates higher confidence) (**B**). An ensemble of structures representing 10 evenly spaced timepoints of a single 20 nanosecond MD simulation. Structures were extracted from the trajectory and rendered as an ensemble in UCSF ChimeraX without alignment, colored by monomer (one monomer in purple, the other in pink) (**C**). The mean relative B-factor was calculated from the MD simulations of the VP3 homodimer (6 independent 20 nanosecond simulations) and plotted on the *y*-axis against the amino acid residue number of two VP3 monomers (**A and B**) concatenated on the *x*-axis. Error bars represent SEM (**D**).

In infected cells, VP3 can exist as a homodimer with the dimerization domain of VP3 predicted to lie within the N-terminus of the protein (25). We therefore generated a predicted structure for the VP3 homodimer using AlphaFold3. The VP3 dimer model featured interactions between the N-termini of the monomers, but by virtue of the flexible hinge regions predicted in the monomer, the central domains wrapped around the N-termini, leading to an overall compact “core” conformation. The C-termini of both subunits extended from the compact cores and exhibited the same low confidence we noted in the monomer model (Fig. 7A and B). MD simulations of the VP3 homodimer revealed the C-termini of both subunits remained highly flexible and dynamic (Movie S2). The resultant trajectories of the alpha carbons in the VP3 homodimer were visualized as an ensemble of 10 evenly spaced timepoints from a single 20 nanosecond MD simulation (Fig. 7C), demonstrating the breadth of the conformation space explored by the C termini. B-factors were calculated for the alpha carbons in the homodimer, and these data indicated that the elevated C-terminal B-factors observed in the monomer simulations were also observed for both subunits of the dimer (Fig. 7D).

### The C-terminal IDR is not required for the recruitment of IBDV VP3 into VFs

**Fig 8:**
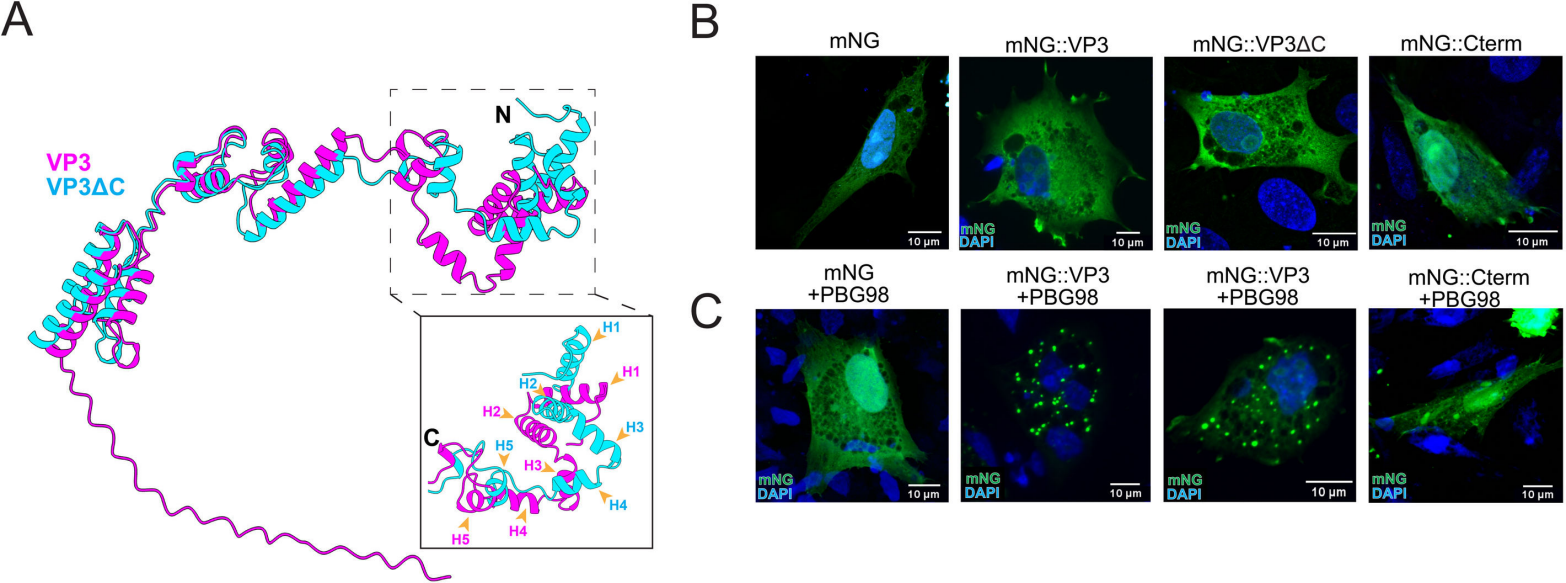
The C-terminal IDR is not required for the recruitment of IBDV VP3 into VFs. AlphaFold3 predictions for the wt VP3 (magenta) and the VP3ΔC (cyan) were aligned with ChimeraX and overlaid (N terminal helices numbered H1–5) (**A**). DF-1 cells were transfected with mNG, mNG::VP3, mNG::VP3ΔC, or mNG::Cterm (**B**) or were co-transfected with the plasmids and simultaneously infected with IBDV strain PBG98 (MOI 5) (**C**). Cells were fixed at 12 hpi, and the nuclei were stained with DAPI (blue), and the mNG signal is shown in green. Scale bars, 10 µm.

We next aimed to compare the behavior of the full-length VP3 with a VP3 molecule that lacked the 36-residue C-terminus. We therefore truncated the mNG::VP3 construct after residue 221 of VP3, to form mNG::VP3ΔC. An alignment of the predicted structures of VP3 and VP3ΔC using Alphafold3 and ChimeraX revealed an identical predicted secondary structure configuration, with differences between the structures contributed only by the flexible linker regions, demonstrating that there were no predicted folding defects in the remaining protein (Fig. 8A). We then transfected DF-1 cells with the mNG::VP3ΔC construct and stained the cells with α-VP3, revealing a complete overlap of the mNG and VP3 signals and a cytoplasmic distribution matching that of full-length VP3. Moreover, colocalization analysis of the mNG::VP3ΔC signal and the anti-VP3 signal revealed a Manders’ coefficient of 0.903 and Spearman’s rank correlation of 0.793 (Fig. S3), demonstrating that truncating the C-terminus did not substantially affect the intracellular localization of the protein in transfected cells, or the binding of the anti-VP3 antibody.

To determine whether deletion of the C-terminus altered the recruitment of exogenous VP3 to VFs, DF-1 cells were transfected with mNG, mNG::VP3, mNG::VP3ΔC, or a construct where the 36 CTR of VP3 was fused to mNG (mNG::Cterm), in the presence or absence of an infection with IBDV strain PBG98 (Fig. 8). The mNG signal was observed to be redistributed from diffusely cytoplasmic to punctate in infected cells transfected with mNG::VP3, mNG::VP3ΔC, or mNG::Cterm, indicating that deletion of the C-terminus did not prevent the recruitment of exogenous VP3 to IBDV VFs, and indicating that the C-terminus itself was also recruited to the VFs. As VP3ΔC contains the core region, which is known to interact with RNA (31), and the C term is known to interact with VP1 (32), we suggest that VP3ΔC is recruited to the VF through interaction with RNA, and that the C term is recruited to the VF through interaction with VP1.

### The presence of VP3ΔC does not significantly impede IBDV replication

Since we had identified that truncation of the VP3 C-terminus did not preclude recruitment of VP3 to VFs formed by infection, we sought to determine whether rescue of viable virus lacking the C-terminus was possible, using our in-house reverse genetics system (33). As VP3 is located at the C-terminus of the polyprotein that is translated from segment A (Fig. 5A), it was possible to remove the C-terminus of VP3 by truncating the segment A coding region by 36 amino acids, to form SegAΔC. DF-1 cells simultaneously transfected with SegAΔC and SegB failed to exhibit any detectable cytopathic effect (CPE) after blind passage, indicating that the absence of the C-terminus prevented the generation of productive virus. We further attempted a rescue in *trans*, by simultaneously transfecting DF-1 cells with SegAΔC, SegB, and VP3, and blind passage was attempted in cells also transiently expressing VP3. However, no CPE was detectable. To determine whether the presence of VP3ΔC acted in a “dominant negative” manner and impeded viral replication, we simultaneously infected and transfected cells with strain PBG98 IBDV alongside mNG, mNG::VP3, mNG::VP3ΔC, and mNG::Cterm, and we measured viral replication by RT-qPCR and TCID_50_ at 7, 16, and 24 h post-infection (hpi). We found no significant difference in viral replication between the infected and co-transfected cells at any timepoint (Fig. S4). Taken together, these data indicate that although a virus encoding VP3ΔC could not be rescued, the presence of VP3ΔC does not significantly impede the replication of wt virus. While it is possible that the truncated SegA RNA might impede the ability to rescue the virus, it might be possible to rescue the virus in a cell line that stably expresses wt VP3, but this was beyond the scope of the present study.

### The IBDV VP3 C-terminal IDR is not required for the formation of cytoplasmic puncta

**Fig 9:**
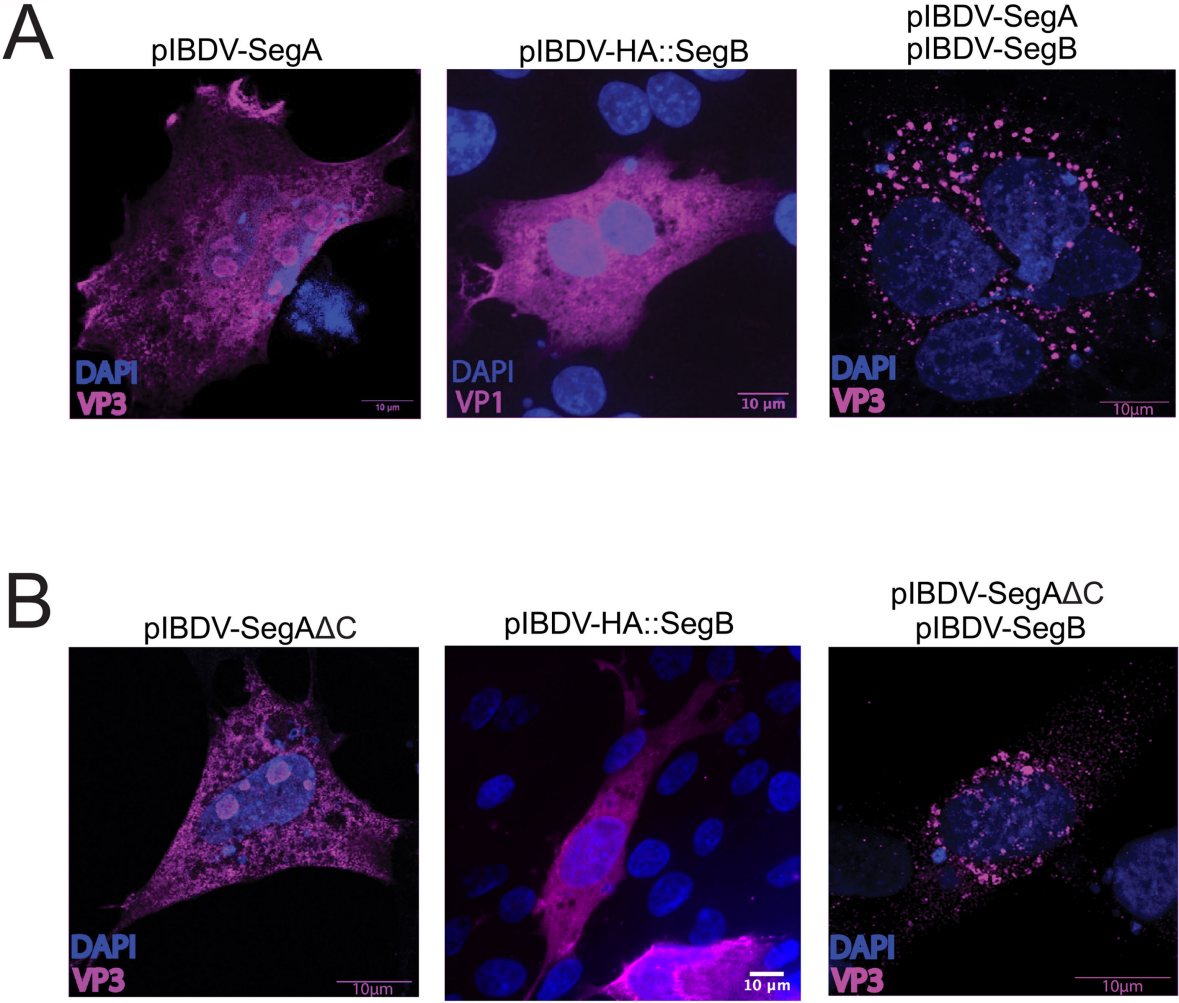
The IBDV VP3 C-terminal IDR is not required for the formation of cytoplasmic puncta. DF-1 cells were either transfected with SegA or HA::SegB and fixed and stained with anti-VP3 or anti-HA antibodies at 18 hpt, or DF-1 cells were simultaneously transfected with both SegA and SegB and fixed and stained with an anti-VP3 antibody at 18 hpt (**A**). DF-1 cells were either transfected with SegAΔC or HA::SegB, and were fixed and stained with anti-VP3 or anti-HA antibodies at 18 hpt, or DF-1 cells were simultaneously transfected with both SegAΔC and SegB, and fixed and stained with an anti-VP3 antibody at 18 hpt. The nuclei were stained with DAPI (blue), and the VP3 or HA-VP1 signals are shown in magenta. Scale bars, 10 µm (**B**).

Although our data indicated that VP3 recruitment to IBDV VFs was independent of the C-terminus, the VFs formed by co-infection and transfection contained a mixture of exogenously expressed VP3ΔC and full-length VP3 derived from the infecting virus, which prevented an analysis of how the VP3 C-terminus affected the formation and properties of the VFs. To address this, we transfected DF-1 cells with SegAΔC and HA::SegB. Similarly to the wt plasmids, cells expressing SegAΔC and HA::SegB alone took on a diffuse cytoplasmic staining pattern, but when cells co-expressed both, cytoplasmic puncta were observed (Fig. 9), demonstrating that the VP3 C-terminal IDR was not an absolute requirement for the appearance of cytoplasmic puncta.

### The IBDV VP3 C-terminal IDR promotes the formation of cytoplasmic puncta and influences their size and shape

**Fig 10:**
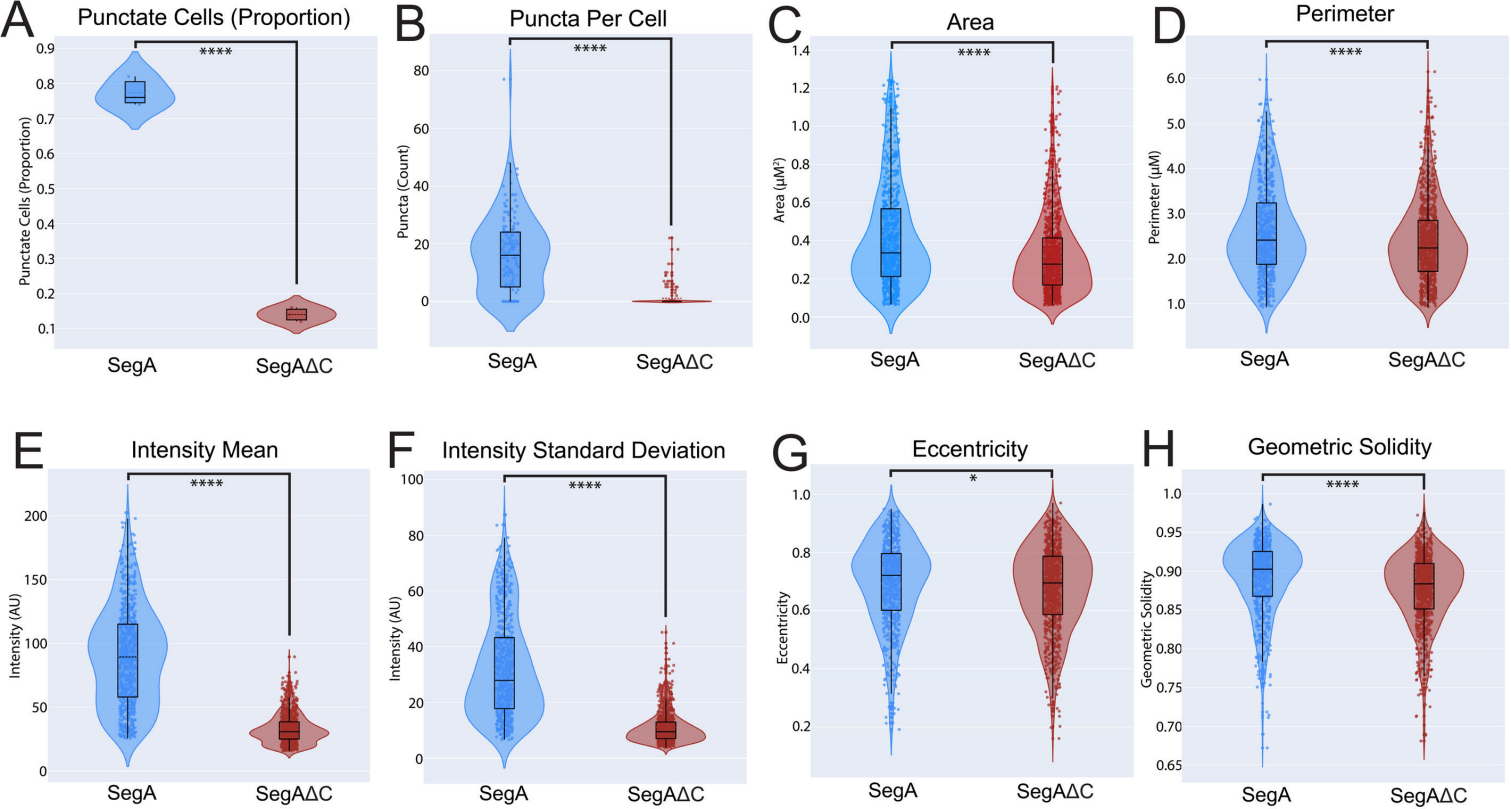
The IBDV VP3 C-terminal IDR promotes the formation of cytoplasmic puncta and influences their size and morphology. DF-1 cells were co-transfected with either SegA and SegB::mNG or SegAΔC and SegB::mNG. From each of 3 replicates, 50 cells were randomly selected at 34 hpt, and their phenotype (punctate or non-punctate) was classified (**A**), and the puncta per cell were counted (**B**). Fifty cells prepared in the same manner were then imaged individually by confocal microscopy, and puncta were segmented by watershed segmentation and the area, perimeter, mean fluorescence intensity, intensity standard deviation, eccentricity, and geometric solidity (a measure of morphological “roundness” or “smoothness”) were measured with the Scikit-image python module (**C–H**).

To evaluate how the VP3 IDR affected the physical properties of the puncta, we modified our SegB plasmid by adding mNG to the C-terminus of the VP1 CDS (SegB::mNG) (Fig. S5). We then transfected cells with either a mixture of SegA and SegB::mNG, or a mixture of SegAΔC and SegB::mNG, and we detected the presence of mNG-positive cytoplasmic puncta that colocalized with the VP3 signal (Fig. S5). Next, 50 randomly selected cells from each of three independent replicates of cells transfected with either SegA and SegB::mNG or SegAΔC and SegB::mNG were imaged, and the puncta were counted. Although cells containing puncta were observed in the absence of the C-terminus, they were detected at a significantly reduced rate (*P* < 0.0001) (Fig. 10A). Furthermore, among the cells that did contain puncta, the mean number of puncta per cell was significantly reduced in the ΔC system compared to wt (*P* < 0.0001) (Fig. 10B). Taken together, these data demonstrate that although the IBDV VP3 C-terminal IDR was not required for the formation of cytoplasmic puncta, it did promote their formation.

Six morphological properties of the puncta were then measured: area, Crofton perimeter, single-puncta mean fluorescence intensity, single-puncta intensity standard deviation, eccentricity, and geometric solidity (a measure of morphological “roundness” or “smoothness”). Puncta that formed in the presence of wt VP3 had a significantly larger average area and perimeter than puncta formed in the presence of VP3ΔC (*P* < 0.0001) (Fig. 10C and D). Moreover, wt VP3 puncta had a significantly greater mean fluorescence intensity and a greater single-puncta intensity variability than VP3ΔC puncta (*P* < 0.0001) (Fig. 10E and F), consistent with the VP3 C-terminal IDR contributing to the size and intensity of the puncta. Finally, wt VP3 puncta had a significantly higher mean eccentricity than VP3ΔC puncta (*P* < 0.05) and exhibited significantly higher mean geometric solidity (*P* < 0.0001), indicating that the VP3 C-terminal IDR contributed to the “roundness” or “smoothness” of the puncta. Taken together, these data demonstrate that the VP3 C-terminal IDR influenced the size and shape of the cytoplasmic puncta.

### The VP3 C-terminus affects the physical properties of VFs

**Fig 11:**
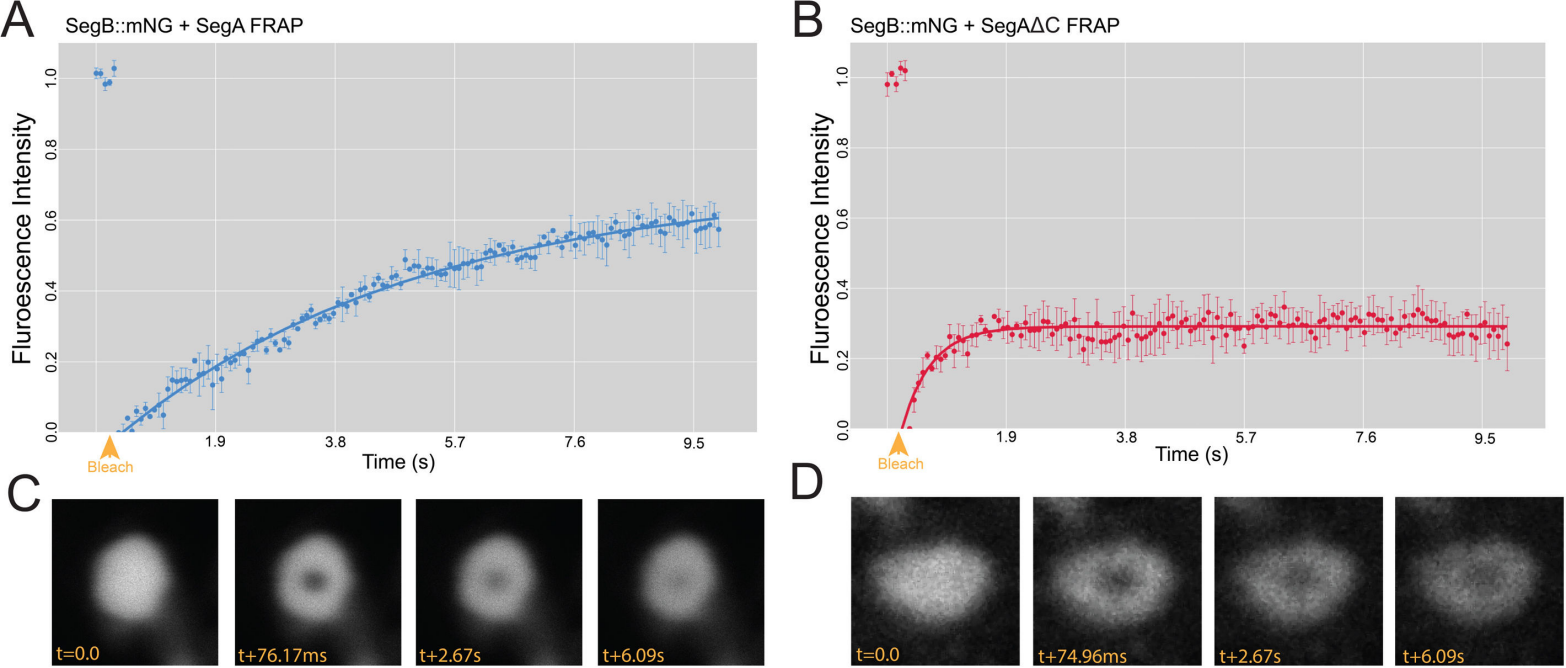
The VP3 C-terminal IDR significantly contributes to the mobile fraction of the molecules within the cytoplasmic puncta. DF-1 cells were co-transfected with a mixture of SegA and SegB::mNG, and a time-lapse series was captured by live confocal microscopy at 14 hpt with a single iteration of point bleaching on the 5th frame (yellow arrowhead). Intensity data were extracted and used to calculate a FRAP recovery curve. Results are from three replicates; error bars = SEM (**A**). DF-1 cells were co-transfected with a mixture of SegAΔC and SegB::mNG, and a FRAP recovery curve was generated in the same way (**B**). Representative recovery images for a photobleached puncta formed in the presence of SegA with the time post-bleach (t) shown in milliseconds (ms) (**C**). Representative recovery images for a photobleached puncta formed in the presence of SegAΔC with the t post-bleach shown in ms (**D**).

Finally, we aimed to characterize differences in the physicochemical properties of puncta formed in the presence of the wt VP3 or the VP3ΔC by performing FRAP. Briefly, we transfected DF-1 cells with either a mixture of SegA and SegB::mNG or a mixture of SegAΔC and SegB::mNG, and we captured time-lapse series by live confocal microscopy at 14 hpt, with a single iteration of point bleaching on the 5th frame of each series. We observed a notable difference between the recovery behavior of puncta formed in the presence of wt VP3 and the VP3ΔC (Fig. 11). Puncta in cells expressing VP3ΔC exhibited a partial recovery in fluorescence after photobleaching, but the mobile fraction was calculated as only 0.291, indicating that only 29.1% of the molecules exhibited any diffusion. This was in sharp contrast to the behavior of puncta formed in cells expressing the wt VP3, where although the recovery half-time was longer at 3.517 s (compared to 0.341 s in the VP3ΔC puncta), the calculated mobile fraction was 0.704, indicating that 70.4% of the molecules exhibited diffusion, a significantly greater proportion compared to the VP3ΔC puncta (*P* < 0.01). These data demonstrate that the VP3 C-terminal IDR affected the physical properties of the VFs. Moreover, we also evaluated the liquidity of the puncta formed in the chicken macrophage cell line HD11 and found similar results: the puncta formed in the presence of wt VP3 exhibited a robust recovery in fluorescence following photobleaching, whereas the puncta formed in the presence of VP3ΔC did not (Fig. S7), demonstrating this phenomenon was not cell type specific.

**Fig 12:**
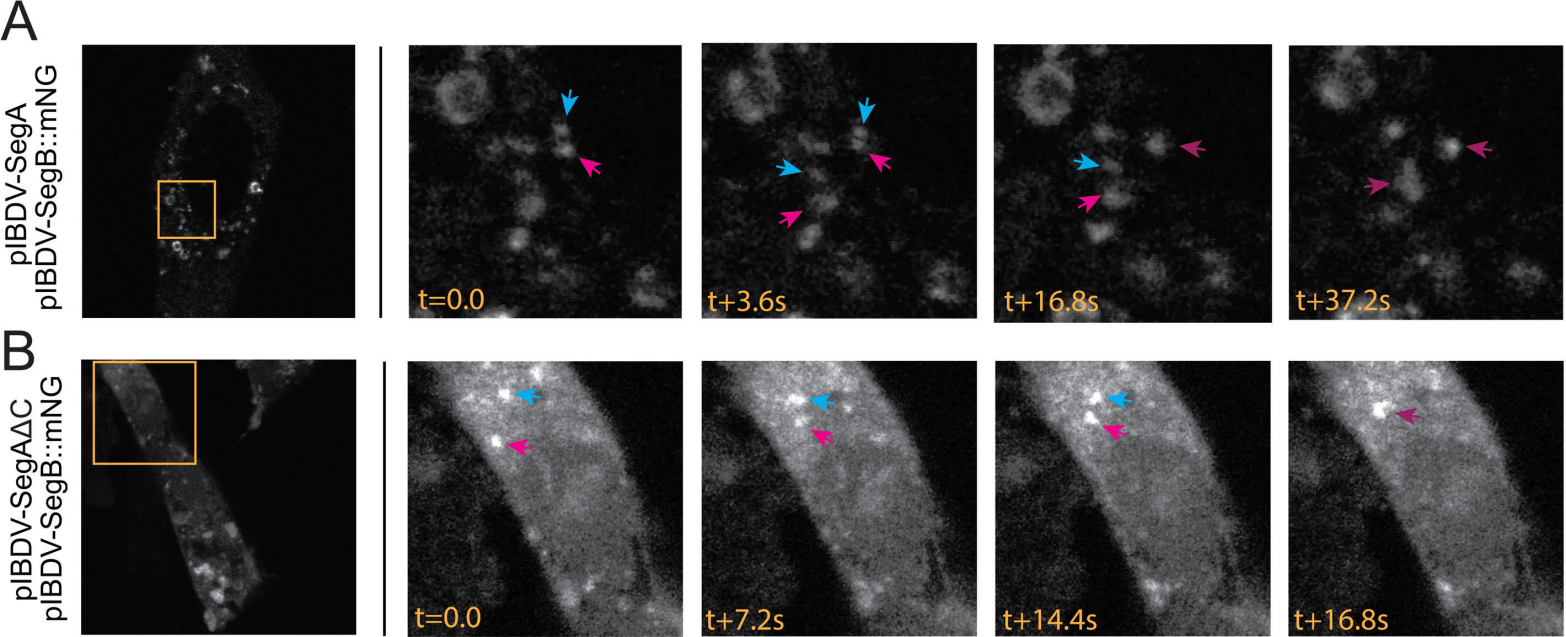
Puncta formed in the presence or absence of the VP3 C-terminal IDR are dynamic. DF-1 cells were transfected with a mixture of SegA and SegB::mNG (**A**), or DF-1 cells were transfected with a mixture of SegAΔC and SegB::mNG (**B**). At 34 hpt, cells were imaged by live-cell confocal microscopy, capturing images every five frames to make a time lapse. Still frames from the timelapses are shown. The leftmost images show whole-cell images (mNG signal shown in white), with stills from the inset regions (orange boxed regions) shown thereafter. The timestamp (t) of imaging is shown in seconds (s). Arrows show individual puncta that undergo fusion.

**Fig 13:**
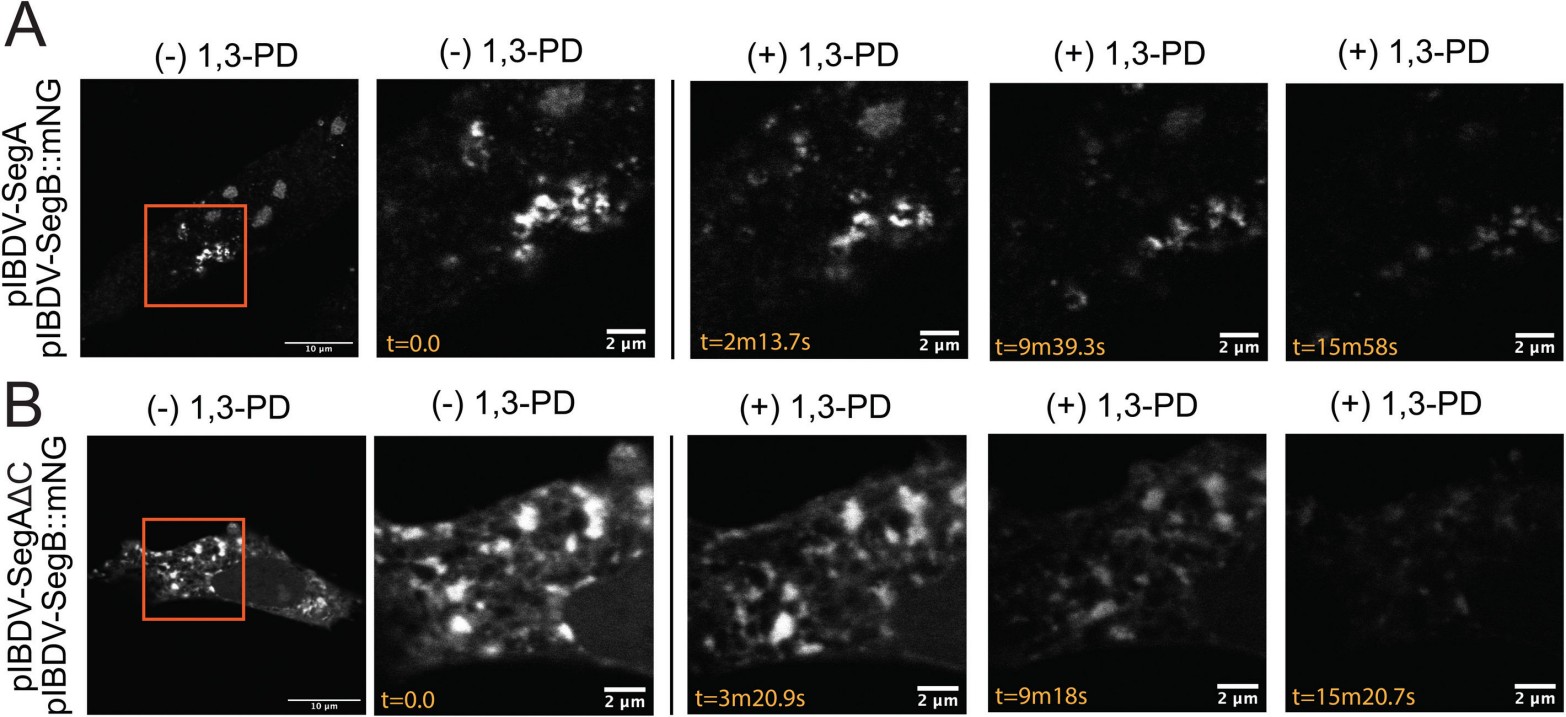
Puncta formed in the presence or absence of the VP3 C-terminal IDR are sensitive to aliphatic diol treatment. DF-1 cells were transfected with a mixture of SegA and SegB::mNG (**A**), or DF-1 cells were transfected with a mixture of SegAΔC and SegB::mNG (**B**). Transfected cells were treated with 1,3-Propanediol (1,3-PD) while capturing time-lapse images. Briefly, cells were initially maintained in media for acquisition, and at time (t) = 0 seconds (s), 1,3-PD was added to achieve a final concentration of 4% (vol/vol). The leftmost images show whole cells (mNG signal shown in white), with stills from the inset regions (orange boxed regions) shown thereafter. The timestamp (t) of imaging is shown in seconds (s).

To assess other behaviors associated with LLPS, we transfected DF-1 cells with either a mixture of SegA and SegB::mNG or a mixture of SegAΔC and SegB::mNG and observed the cells by live-cell confocal microscopy, capturing images every five frames to make a time lapse. Interestingly, we observed that both the cytoplasmic puncta made in the presence of either wt VP3 or VP3ΔC were mobile, moving throughout the cytoplasm on similar, rapid timescales, and underwent fusion events (Fig. 12), indicating that these key liquid-like properties were retained in the absence of the VP3 C-terminus. Furthermore, we characterized the response of the puncta to treatment with small aliphatic diols, which have been observed to disrupt LLPS structures (34). Briefly, we transfected DF-1 cells with either a mixture of SegA and SegB::mNG or a mixture of SegAΔC and SegB::mNG, and treated the transfected cells with 1,3-propanediol (1,3-PD), an aliphatic diol that exhibits lower toxicity than the more commonly used 1,6-hexanediol (1,6-HD), while capturing time-lapse images (9). Although the toxicity of 1,3-PD was reduced as compared to 1,6-HD, eventually the cells did succumb to the treatment, meaning our observations were limited to approximately 10 min post-treatment where we could reliably image the puncta. Over the course of the time lapse, 1,3-PD caused a moderate degree of disruption to puncta made with both wt VP3 or VP3ΔC (Fig. 13). The margins of most puncta exhibited a loss of definition, although complete dissolution was only observed for a subset of smaller puncta. Both puncta formed in the presence of wt VP3 and in the presence of VP3ΔC were sensitive to diol treatment, but the responses varied too widely between cells and between puncta within a single cell to quantitatively distinguish differences in the behavior of the puncta made with either wt VP3 or VP3ΔC.

## DISCUSSION

In previous work, we determined that the VFs of birnaviruses such as IBDV were formed through the process of LLPS (10, 19), but the molecular mechanism of how birnaviruses drive LLPS to form their VFs remained unknown. As a major component of IBDV VFs is the VP3 protein (10, 18, 23), we sought to investigate its role in forming VFs. In previous work, we expressed eGFP::VP3 in cells and observed cytoplasmic puncta (10). However, in the present study, when we expressed untagged VP3 independently in cells, we did not observe puncta characteristic of VFs, suggesting that VP3 alone was insufficient to drive phase separation. To reconcile these apparently conflicting results, in the present study, we investigated fusing VP3 to different tags. This revealed that eGFP::VP3 formed cytoplasmic puncta similar to our previous observation, but when VP3 was tagged to other molecules, these puncta were not apparent, and the distribution more resembled that of the untagged VP3. Therefore, we concluded that VP3 does not form puncta in cells when expressed alone, and the nature of the tag used is important in determining the cellular distribution of VP3. Furthermore, we removed the VP3 gene from our reverse genetics system and demonstrated the absence of cytoplasmic puncta even when all other viral proteins and vRNA were expressed. Moreover, when this system was supplemented with exogenous wt VP3, the punctate phenotype was recovered. Taken together, these data demonstrate that VP3 is necessary, but not sufficient, for the formation of IBDV VFs. This is in contrast to the scaffold protein of ReoV (µNS), which spontaneously phase separates in transfected cells in the absence of other viral components (15).

Interestingly, VP3 has been reported to form small foci in QM-7 cells stably expressing the protein (23). However, these foci did not resemble the smooth or round structures that are characteristic of LLPS, and the authors did not assess their physical properties. In the present study, we also observed some small foci in DF-1 cells expressing VP3 alone. These formed what we would describe as a granular appearance and were particularly notable after fixation with PFA (e.g., see Fig. 3, VP3). However, these foci did not have the same appearance as bona fide VFs and were too small to meaningfully assess liquid-like recovery by FRAP. It is known that VP3 interacts with the cytoplasmic leaflet of endosomal membranes by binding phosphatidylinositol-3-phosphate (PI3P) (23, 35, 36), and it is possible that accumulation of VP3 along these membranes is responsible for the granular appearance we observed in DF-1 cells, and the foci that have previously been reported in QM-7 cells. Moreover, in some cells, we also observed a ring-like “annular” phenotype when VP3 was expressed alone (Fig. S6), which might also be consistent with VP3 interacting with intracellular membranes.

Next, we sought to define the minimal component set for IBDV phase separation. Although we were able to detect some punctate structures in cells simultaneously expressing VP3 and VP1, these showed limited recovery by FRAP. It was only when we simultaneously expressed VP3 with IBDV Segment B (which encodes VP1 protein and provides a template for VP1-driven vRNA synthesis) that we observed robust, liquid-like FRAP recovery, indicating that the minimal component set for phase separation comprised VP3 and VP1, and may also include vRNA. VP3 is known to bind RNA via its core region, and VP1 via its C-terminus (31, 32), and VP1 is known to bind vRNA and act as a VPg-like cap, as well as being the polymerase that synthesizes vRNA. We therefore propose that VP3 bridges VP1 and vRNA via its different domains to form a higher-order complex that phase separates. It should be noted, however, that definitively establishing the necessity of vRNA for phase separation was beyond the scope of the present study and would require inhibiting vRNA production, for example, by VP1 active site mutation or chemical inhibition with ribavirin, and then demonstrating that condensate formation is inhibited.

Having identified the critical role of VP3 in the formation of IBDV VFs, we used Alphafold modeling and MD simulations to robustly characterize the behavior of the predicted structure of the IBDV VP3 monomer and homodimer. We analyzed the predicted thermal motion (B-factor) and internal positional correlation of the atoms across the structure, and based on these data, we concluded that the 36 C-terminal amino acids of VP3 comprised a predicted highly dynamic IDR, a result that agreed with the predicted behavior of this region based on the protein sequence. IDRs are frequently implicated as drivers of LLPS, as their conformational plasticity is well-suited to the multivalent weak interactions that cause the phenomenon (37). We therefore hypothesized that VP3 acts as a scaffold for IBDV VFs, with its IDR as a key driver of LLPS. Consistent with this hypothesis, we discovered that the rate of cytoplasmic puncta formation was significantly reduced in the presence of VP3 lacking the C-terminus, as compared to the presence of full-length wt VP3, with fewer punctate cells being observed and fewer puncta per cell. Moreover, the puncta that did form in the absence of the VP3 C-terminus were significantly smaller, had a reduced mean fluorescence intensity, and were less “round” or “smooth” than those formed from wt VP3. Additionally, using FRAP, we discovered that the mobile fraction in puncta that formed in the absence of the VP3 C-terminus was significantly lower (0.291) than the puncta that formed in the presence of full-length wt VP3 (0.704) (*P* < 0.01), demonstrating that the VP3ΔC puncta were less liquid-like. Taken together, these data demonstrated that the C-terminal IDR of VP3 was a potent driver of cytoplasmic puncta formation and influenced their liquidity.

Interestingly, removal of the VP3 C-terminus did not completely abolish cytoplasmic puncta formation, suggesting that the VP3 C-terminal IDR was not strictly required for puncta formation. We also observed that exogenous VP3 could be recruited to IBDV VFs in infected cells, even in the absence of the C-terminus. Such behavior precluded the C-terminus from being a requisite for VF compatibility. Moreover, as VP3 is known to dimerize, it is possible that when VP3ΔC was recruited to the VFs of infected cells, it may have formed heterodimers with full-length VP3 provided by the virus. LLPS requires multivalent intermolecular contacts, frequently three or more (37), and although the two protruding C-termini of the dimer were appealing candidates as contact sites to ensure multivalency associated with LLPS, the ability of the VP3ΔC to be recruited to VFs in infected cells suggested that deletion of at least one C-terminus did not remove this required valency. Therefore, we hypothesize that the VP3 C-terminal IDR might interface with other client biomolecules, thus influencing the physical properties of the resultant structures, for example, by affecting the kinetics of the recruitment of biomolecules to VFs, or the packing and diffusive environment within the structures. Furthermore, the interaction of the VP3 C-terminus with VP1 is known to substantially promote VP1’s polymerase activity, leading to increased vRNA synthesis (38). As we have demonstrated that vRNA is required for the formation of puncta with liquid-like properties, the VP3 C-terminus may also influence the liquidity of VFs by promoting vRNA synthesis. Simply put, the VP3 C-terminal IDR could be important in modulating how liquid the VFs are.

Interestingly, we observed minimal differences in the dynamic behaviors of the puncta formed in the presence of full-length wt VP3 and in the presence of VP3ΔC: both were freely mobile in the cytoplasm and appeared to undergo fusion events, and both were sensitive to treatment with small aliphatic diols, which are means of designating LLPS (34). It remains unknown why some properties of the puncta were more significantly altered by the removal of the VP3 C-terminal IDR than others. However, as these live-cell imaging assays were applied qualitatively in our study, it was not possible to draw quantitative conclusions as to how the presence or absence of the VP3 C-terminus affected these behaviors. In addition, as the aliphatic diols broadly perturb the hydrogen bonding environment of the entire cell, they caused a notable toxicity in DF-1 cells, even at low concentrations. To mitigate cytotoxicity, we treated cells with 1,3-PD, a less-toxic compound in the same class as the more commonly used 1,6-HD; however, responses varied too widely between cells and between puncta within a single cell to quantitatively distinguish differences in the behavior of puncta formed in the presence of the full-length VP3 or VP3ΔC, which is a limitation of our study.

The most quantitative assay that measured the physical properties of the puncta was FRAP, and as mentioned above, these data were consistent with the VP3 C-terminus promoting the liquidity of the puncta, as the mobile fraction measured for puncta formed in the presence of the full-length VP3 was significantly higher than in the puncta formed by VP3ΔC. Interestingly, we also observed that the recovery of the VP3ΔC puncta exhibited a shorter recovery half-life (0.341 s), whereas the recovery of the wt VP3 puncta was longer (3.517 s). Although both were very rapid in the context of FRAP, a faster recovery half-life did not intuitively pair with a substantially reduced mobile fraction, as rapid recovery is suggestive of liquidity, whereas a reduced mobile fraction is suggestive of rigidity. However, it is important to note that in our system, the mNG fluorophore was fused to the client protein VP1, rather than the scaffold protein VP3. Previous studies have indicated that VP3 directly binds VP1 via the 10 C-terminal amino acids of VP3 (32). As these are absent in the ΔC system, it is therefore possible that the unexpectedly rapid recovery half-life in the VP3ΔC puncta was due to the presence of a small amount of unbound VP1-mNG that was able to move more readily as it was not tethered to VP3, even if the rest of the puncta was more rigid. However, further work is needed to confirm this effect.

**Fig 14:**
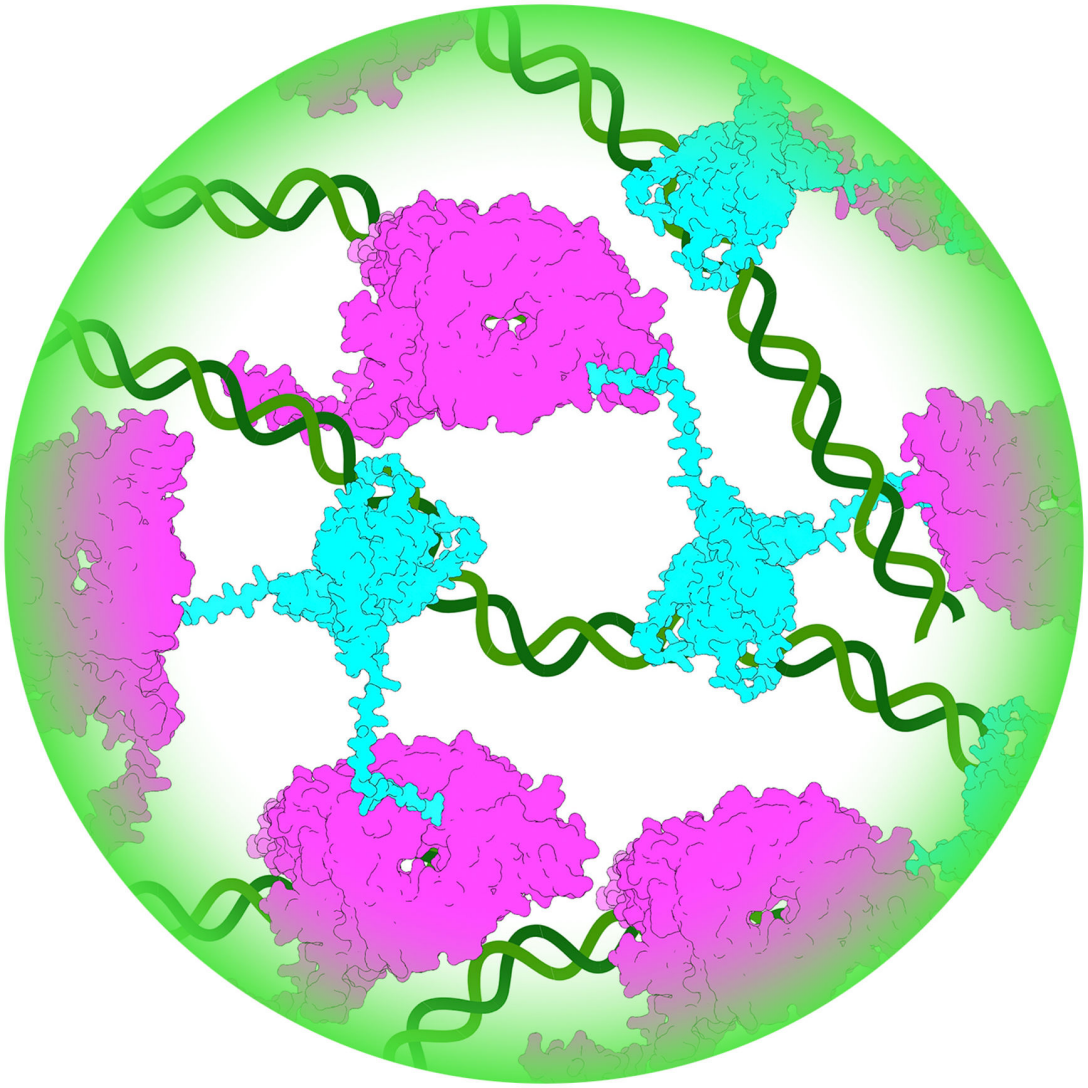
Schematic of the model for how VP1, VP3, and vRNA interact in a phase-separated biomolecular condensate. VP3 (blue) interacts with RNA (green) via the core domain, and with VP1 (magenta) via the C-terminus. Interaction of VP3 with VP1 increases the synthesis of dsRNA by the polymerase, which contributes to the growing condensate.

In summary, our data suggest that IBDV VP3 forms a higher-order matrix with VP1 and potentially vRNA that collectively drive LLPS to form biomolecular condensates. Moreover, we discovered that the 36 C-terminal amino acids of IBDV VP3 comprised a predicted IDR that promoted the formation of the condensates and modulated their liquidity. As VP3 is thought to bind RNA via the “core” region (31), and VP1 via the C-terminus (32), our working model is that VP3 is recruited to the VFs by interacting with the RNA via the core, and then modulates the liquidity of the VFs by interacting with VP1 and potentially other client molecules via the C-terminal IDR. Moreover, as interaction of VP3 with VP1 is known to increase the synthesis of dsRNA by the polymerase (38), this could contribute to the growing condensate (Fig. 14). This is somewhat analogous to rotavirus NSP2, where the flexibility of the CTR supports LLPS and the biogenesis of rotavirus VFs (17). Moreover, a single point mutation (K294E) in the NSP2 CTR was predicted to reduce its flexibility. Compared to wild-type NSP2, this mutation induced smaller and more numerous viroplasms in infected cells, severely impacted viral replication, and adversely affected condensation behavior with NSP5 *in vitro (17*). These data demonstrate the sensitive dependence of rotavirus viroplasm condensation dynamics and properties on the flexibility of the NSP2 CTR and showed that even small changes to critical protein regions can induce measurable effects on biomolecular condensates that directly impact viral replication. Our data indicate that the C-terminus of IBDV VP3 is likewise an important LLPS-modulating region, and it would be interesting to identify if critical features and residues can act as determinants of VF properties. Furthermore, VP3 is known to bind the cytoplasmic surface of early endosomes via the core (23) and is thought to interact with newly synthesized VP2 capsid proteins via the C-terminus (25), and additional work is necessary to study how these interactions affect LLPS to make further conclusions.

## MATERIALS AND METHODS

### AlphaFold

Structural modeling with AlphaFold2 was accomplished using the AlphaFold2 Colab notebook published by DeepMind (https://colab.research.google.com/github/deepmind/alphafold/blob/main/notebooks/AlphaFold.ipynb?pli=1), based on the amino acid sequence of the VP3 protein from IBDV strain PBG98, obtained from GenBank accession MT010364. Processing was performed using the monomer model, with relaxation performed in GPU mode. Structural modeling with AlphaFold3 was accomplished using the AlphaFold Server (https://www.alphafoldserver.com). Structures were visualized with UCSF ChimeraX v.1.17.1 (UCSF, San Francisco, CA) (39).

### Predicted aligned error

AlphaFold’s predicted error in the relative position of each pair of residues, measured in Angstroms, was extracted from the .zip archives generated by the AlphaFold Colab notebook by ingesting the “predicted_aligned_error.json” file with Python’s JSON module, and values were plotted as a heatmap with Plotly (Plotly Technologies Inc.), see supplemental material for more information.

### IUPred3 disorder plot

IUPred3 was accessed through the Eötvös Loránd University web portal (https://iupred3.elte.hu/) and run in short disorder mode with medium smoothing. Results were downloaded in JSON format and loaded using Python’s JSON module and plotted as a line plot with Plotly.

### Sequence entropy

The sequence entropy of VP3 was determined and plotted as a line plot with Plotly (see supplemental material for more information).

### MD simulations

To run the MD simulations and generate movies, the software GROMACS was installed on a fresh installation of Ubuntu 22.04, running on an AMD 3800X processor with 32GB of RAM. The GROMACS binary was compiled with the (-DGMX_GPU = ON) flag, with CUDA cores provided by an Nvidia GTX 1080Ti graphics coprocessor (Nvidia, CA). Each simulation was 20 nanoseconds in duration and performed using the OPLS-AA/L all-atom force field with a 2 femtosecond (fs) timestep. Energy minimization and equilibration were carried out before each simulation. The trajectories of every alpha carbon atom in VP3 were determined, and the absolute point positions were extracted from the trajectory data. Periodic boundary condition effects were corrected algorithmically (see supplemental material for more information), and the alpha carbons of VP3 were visualized as animated three-dimensional scatter plots of the atomic coordinates.

### B factor calculations

The B factor was calculated from the MD simulations (see supplemental material for more information), and the mean and standard error of the mean (SEM) were plotted as a line plot with Plotly.

### Atomic motion correlation analysis

For each MD simulation, the Pairwise Pearson’s product-moment correlation coefficients were calculated for the fluctuations of all alpha carbons about their mean positions (see supplemental material for more information).

### Cell culture

The chicken DF-1 fibroblast cell line (40) was obtained from the American Type Culture Collection (ATCC) (VA, USA), and the HD11 cell line was a kind gift from Dr. Mark Parcells, University of Delaware. Cells were maintained in Dulbecco’s modified Eagle’s medium (DMEM) (ThermoFisher Scientific, MA, USA) supplemented with 10% heat-inactivated fetal bovine serum (FBS) (Gibco, ThermoFisher Scientific, MA, USA) and incubated in an atmosphere of 37°C and 5% CO_2_.

### Plasmids

Plasmids expressing the following were used in this study: IBDV VP3 (untagged), mNG, IBDV VP3 fused to mNG, TC, or eGFP reporters at the N-terminus (mNG::VP3, TC::VP3, eGFP::VP3), IBDV VP3 lacking the C-terminus fused to mNG at the N-terminus (mNG::VP3ΔC) IBDV VP3 C-terminus fused to mNG at the N-terminus (mNG::Cterm), IBDV VP1 fused to FLAG at the C-terminus (VP1::FLAG), IBDV reverse genetics (RG) plasmid encoding segment A (SegA) (19), IBDV RG plasmid encoding segment B (SegB) (19), segment A lacking VP3 (SegAΔVP3), segment A lacking the C-terminus (SegAΔC), segment B with an HA tag inserted between the 5′UTR and the coding region of VP1 (HA::SegB), and segment B with mNG fused at the C-terminus (SegB::mNG). Each of the inserts was cloned into the pSF-CAG-KAN vector (Sigma-Aldrich, DE), and the IBDV sequences of segment A, B, VP1, and VP3 were designed based on the cell-culture-adapted strain, PBG98 (19) (GenBank accession numbers MT010364 and MT010365).

### Molecular cloning

The vector and insert DNA were digested with appropriate restriction nucleases (New England Biolabs [NEB], MA, USA), in accordance with manufacturer protocols for each enzyme. The reactions were then separated by gel electrophoresis, and the Monarch Gel Extraction kit (NEB) was utilized to extract DNA from gel slices. The DNA was quantified by nanodrop, and the backbone and insert DNA were ligated with T4 DNA ligase (ThermoFisher Scientific), in accordance with the manufacturer’s protocols, at 16°C overnight. The ligation product was then used to transform chemically competent *E. coli* (NEB), according to the manufacturer’s protocols. After transformation and outgrowth in SOC media (ThermoFisher Scientific), the mixture was streaked onto agar plates with the appropriate antibiotic selection marker (ThermoFisher Scientific). Plates were incubated at 37°C overnight to allow colonies to develop. Colonies were selected and grown overnight in LB broth (ThermoFisher Scientific), in the presence of selection antibiotic, at 37°C with shaking at 200rpm. A 1 mL aliquot of the broth was reserved from each of these outgrowths, and the remainder was processed using the Spin miniprep kit (Qiagen, MD), and screened by an analytical restriction digest. Once a positive sample was identified, additional LB broth supplemented with the appropriate antibiotic selection was inoculated with the 1 mL reserved aliquot of the positive outgrowth and incubated overnight at 37° with shaking at 200 rpm and processed using the Spin Maxiprep kit (Qiagen), with final elution in molecular biology grade water. The DNA was quantified by nanodrop, diluted with molecular biology grade water to 1,000 ng/µL, and stored at −20°C.

### Transfection

DF-1 cells were transfected with the relevant plasmids using Lipofectamine 2000 transfection reagent (ThermoFisher Scientific), according to the manufacturer’s recommendations for the size of the culture vessel. Briefly, the lipofectamine 2000 reagent and DNA were diluted in Opti-MEM media (Gibco, ThermoFisher Scientific), mixed, and incubated for 20 min at room temperature (RT) before application to adherent cells dropwise into the media.

### Virus

A molecular clone of the cell-culture-adapted IBDV strain PBG98 was rescued by transfecting DF-1 cells with the RG plasmids Segment A and Segment B, and monitoring cells for CPE as previously described (19). The resulting virus was titrated in DF-1 cells and the titer quantified by the tissue culture infectious dose-50 (TCID_50_) method described by Reed and Muench (41). Unless otherwise stated, DF-1 cells were infected at an MOI of 5 in downstream experiments.

### Immunofluorescence microscopy

Twelve-well tissue culture-treated plates (Corning, AZ) were populated with 18 mm no. 1 glass cover slips and seeded with DF-1 cells and maintained as described (see Cell Culture). Cells were washed with sterile phosphate-buffered saline (PBS), fixed in 4% (vol/vol) paraformaldehyde (PFA) in PBS for 15 min at RT, and washed again with PBS. Cells were then permeabilized with 0.1% (vol/vol) Triton-X 100 in PBS for 15 min and incubated with a blocking buffer of 4% (wt/vol) of bovine serum albumin in PBS for 1 h at RT. The primary antibody was then diluted in blocking buffer, and cells were incubated in this solution at RT for 1 h with rocking. After incubation, the primary antibody solution was aspirated, and the cells were washed with PBS. The secondary antibody conjugated to a fluorophore was then diluted in blocking buffer, and cells were incubated in this solution for 1 h at RT with rocking. Cells were washed with PBS, counterstained with DAPI, and cover slips were mounted onto slides prior to imaging.

### Microscopy and image processing

Fluorescence micrographs were acquired with either a Nikon Ti-2 widefield fluorescent microscope with a 60× oil immersion objective, a Zeiss LSM980 scanning confocal microscope with a 63× oil immersion objective, or a Leica Stellaris 8 scanning confocal microscope with a 63× oil immersion objective. To capture mosaic images with the Leica Stellaris 8, a grid of evenly spaced focal points was established manually, and the tiled acquisition and mosaic assembly proceeded automatically via the integrated LAS X software suite, with mosaics exported as .TIF files. Images of individual cells were obtained by cropping the mosaic images directly produced by the LAS X software. Image processing (cropping, scale bars, pseudocolor) was performed in Fiji.

### Puncta quantification and measurement of morphology

Puncta were quantified by acquiring mosaic images with a Leica Stellaris 8 scanning confocal microscope. Cells were selected at random from the fields, and puncta were counted manually. Fifty cells were analyzed for each of three independent trials for each condition. Measurement of puncta morphology in cells was accomplished by automatic segmentation. Briefly, images of 50 single cells were captured with a Zeiss LSM980 Airyscan scanning confocal microscope and processed using a custom Python script, which performed watershed segmentation to isolate puncta. Segmentation was manually reviewed prior to measurement. The script is available on GitHub (https://github.com/LadInTheLab/vp3-llps), and the algorithm utilizes the Scikit-image library for segmentation and measurement. Statistics were calculated by grouping puncta by condition (wt or ΔC) and after a Shapiro-Wilk test determined that the assumption of normality was not valid, a Mann-Whitney *U* test was performed between the groups for each metric.

### Live-cell imaging

Cells were seeded in confocal dishes with 20 mm no. 1 glass bottoms (MatTek, MA) at a density of 2 × 10^6^ cells per dish. Cells were imaged in a live-cell imaging chamber of a Zeiss LSM 980 laser scanning confocal microscope and maintained in a 5% CO_2_ environment at 37°C during imaging. The microscope was configured to minimize laser power (and thus photobleaching) without introducing excess noise, and with Z-drift compensation configured to run every five frames to maintain a constant imaging plane.

### Aliphatic diol treatment

DF-1 cells were seeded in glass-bottom confocal dishes with 20 mm No. 1 glass bottoms at a density of 2 × 10^6^ cells per dish. Immediately prior to imaging, the medium was removed from the cells and 100 µL of fresh media was added. Cells were imaged with a Zeiss LSM 980 laser scanning confocal microscope configured to minimize laser power (and thus photobleaching) without introducing excessive noise, and with Z-drift compensation configured to run every five frames. An open-ended time-lapse acquisition was then configured. Thirty seconds after acquisition began, 900 µL of media containing 4.44% (vol/vol) of the specified aliphatic diol (Beantown Chemical, NH) was carefully added to the dish by pipette, for a final concentration of 4% (vol/vol), with continual imaging. Image acquisition was continued until photobleaching, cell drift, or toxicity became excessive.

### Fluorescence recovery after photobleaching

FRAP was performed using a Zeiss LSM 980 laser scanning confocal microscope. The imaging area was cropped to maximize the proportion of the scanned area occupied by the puncta of interest, and a circular measurement region of interest (ROI) was established, centered about a point ROI. The system was configured to capture a 256 × 256 pixel image every 75.95 milliseconds with a 1AU pinhole. The experiment was configured to capture five pre-bleach frames, followed by a single-frame 488 nm bleaching pulse at 10% power at the point ROI, followed by continuous acquisition of frames thereafter. Extraction of intensity data was performed in Fiji (v2.3.0/1.53f, open source). Unmodified files from the Zeiss microscope (.czi files) were loaded with BioFormats importer, and 3 ROIs were established on the images: “Bleach,” an ROI encompassing the region bleached in the first frame after the bleaching pulse, “puncta,” an ROI encompassing the entire puncta, and “background,” an ROI outside the puncta. The multi-measure function was then used to capture mean intensity data for each ROI across every frame of the experiment, the results of which were exported as comma-separated value (.csv) files for analysis. This analysis was performed in Python, with a script available at https://github.com/LadInTheLab/vp3-llps. The script was based on easyFRAP-web (42). For more information, please see the supplemental material.

### Statistics and plotting

Statistics and plotting were performed in Python unless otherwise specified. Statistical calculations were performed using the SciPy package (43), and plotted using the Plotly package (44).

## Data Availability

Any materials and data that are reasonably requested by others are available from a publicly accessible collection or will be made available in a timely fashion to members of the scientific community for noncommercial purposes.

## SUPPLEMENTAL MATERIAL

Supplemental Material mbio.03107-25-s0001.docx Figures S1 to S7, legends for Movies S1 and S2, and supplemental methods.

Movie S1 mbio.03107-25-s0002.mp4 Animated 3D scatter plot representing the positions of the alpha carbons across a single 20 nanosecond molecular dynamics simulation of the Alfafold2-derived structure of the VP3 monomer.

Movie S2 mbio.03107-25-s0003.mp4 Animated 3D scatter plot representing the positions of alpha carbons across a single 20 nanosecond molecular dynamics simulation of the Alphafold3-derived structure of the VP3 homodimer.

ASM does not own the copyrights to Supplemental Material that may be linked to, or accessed through, an article. The authors have granted ASM a non-exclusive, world-wide license to publish the Supplemental Material files. Please contact the corresponding author directly for reuse.
