# Supplementary material for: The C-terminus of infectious bursal disease virus VP3 encodes a predicted intrinsically disordered region, which promotes the formation of cytoplasmic puncta and modulates their physical properties
Source: mBio. 2026 Jan 12;17(2):e03107-25. doi: 10.1128/mbio.03107-25 (PMC12893007; doi:10.1128/mbio.03107-25)
Supplement: Supplemental Material — Figures S1 to S7, legends for Movies S1 and S2, and supplemental methods. [file mbio.03107-25-s0001.docx]

**Supplemental Material**

**Supplemental Figures.**

**
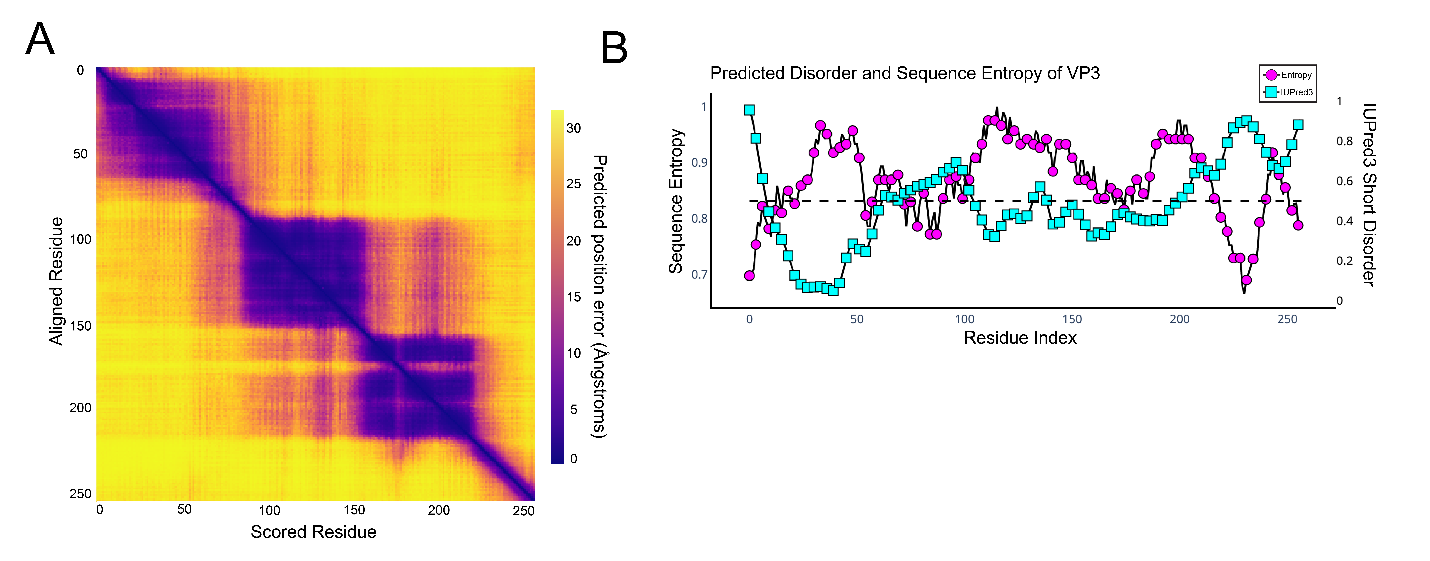
**

**Fig. S1.** A heatmap of the predicted aligned error (PAE) values for the AlphaFold2 prediction of the VP3 monomer. Values represent AlphaFold’s predicted error in the relative position of each pair of residues, measured in Angstroms and plotted on the x and y axes (yellow depicting high error and purple depicting low error) (A). Sequence analysis of VP3 Shannon Entropy (magenta) as measured for a dynamic 20-residue window centered on each residue, compared with the IUPred3 Short Disorder plot (blue). The horizontal dashed line represents an IUPred3 Short Disorder of 0.5 (B).


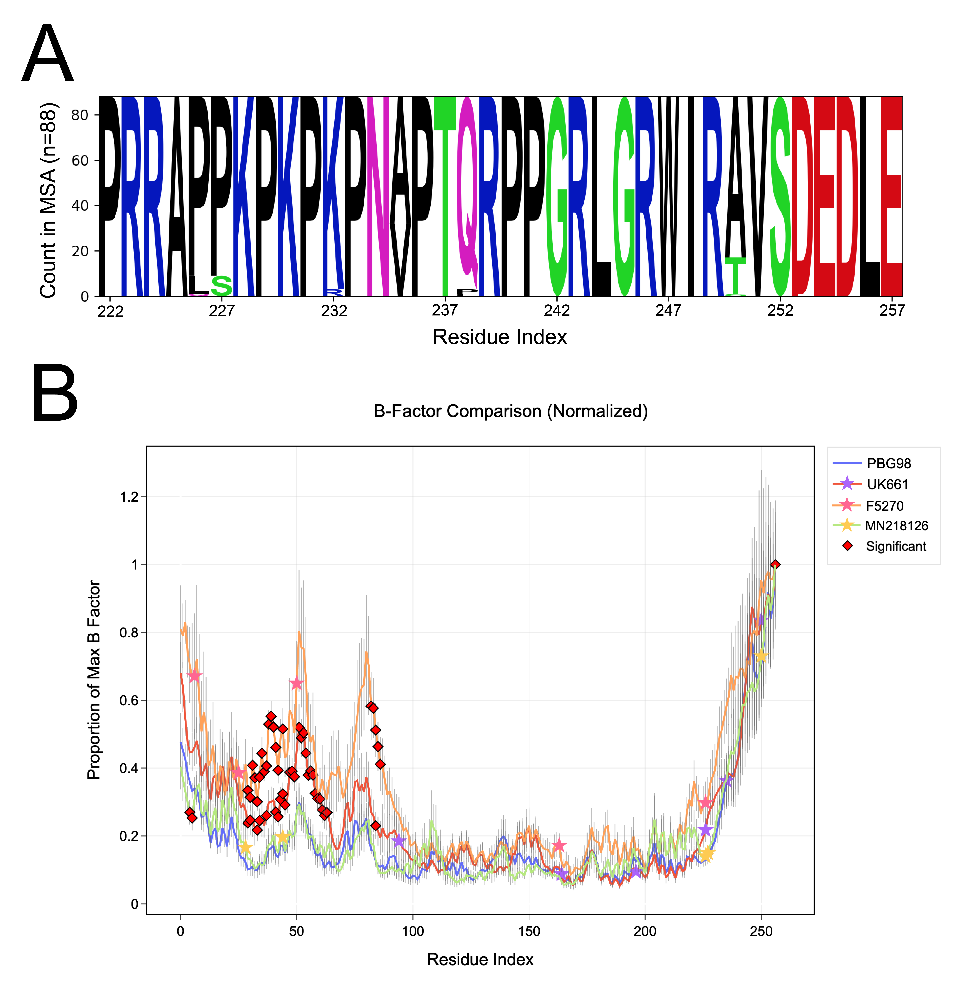


**Fig. S2.** 88 sequences for IBDV VP3 were obtained from GenBank and aligned as a multiple sequence alignment using SnapGene. The 36-residue C-terminal region from each sequence was isolated and processed into a “sequence logo” using the Logomaker python package (A). The structures of VP3 for four different strains of IBDV (PBG98, UK661, F5270, and MN218126) were predicted with AlphaFold2 and monomeric molecular dynamics simulations were performed. Mean relative B-factor, calculated from MD simulations of the VP3 monomer (6 independent 20 nanosecond simulations), was plotted on the y axis against VP3 amino acid residue number on the x axis. Error bars represent standard error of the mean (SEM). Amino acid positions where each strain differs from PBG98 are indicated with a colored star, and positions where B factor differs significantly (p<0.05) from PBG98 are indicated with a red diamond (B).


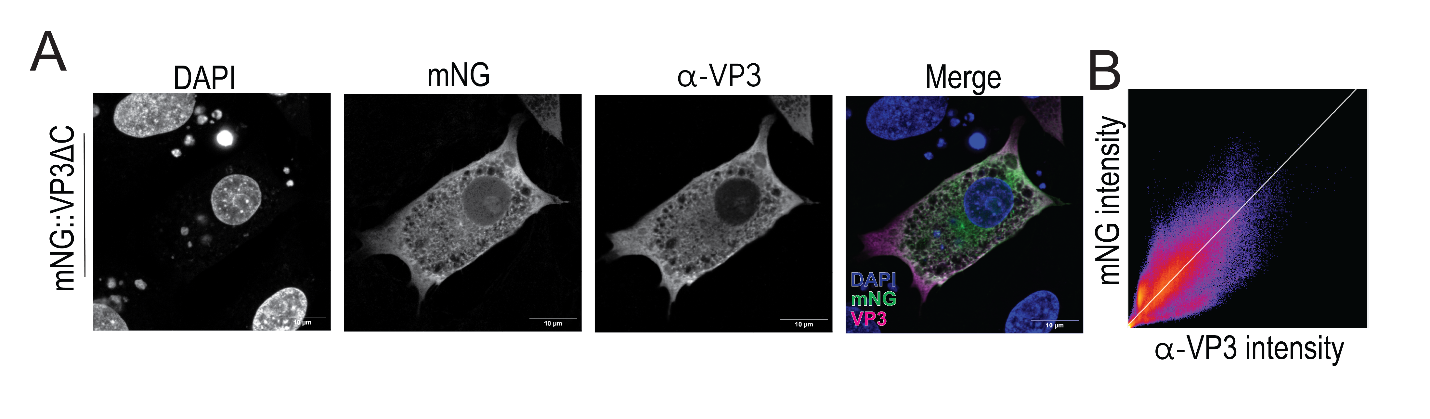


**Fig. S3.** DF-1 cells were transfected with mNG::VP3$\Delta$C, fixed 18hpt, and immunostained with an antibody raised against full-length VP3 (nuclei stained with DAPI). The signal from individual channels is shown in black and white, with the merged image in color) (A). Colocalization analysis of the mNG::VP3$\Delta$C signal and the anti-VP3 signal (B).


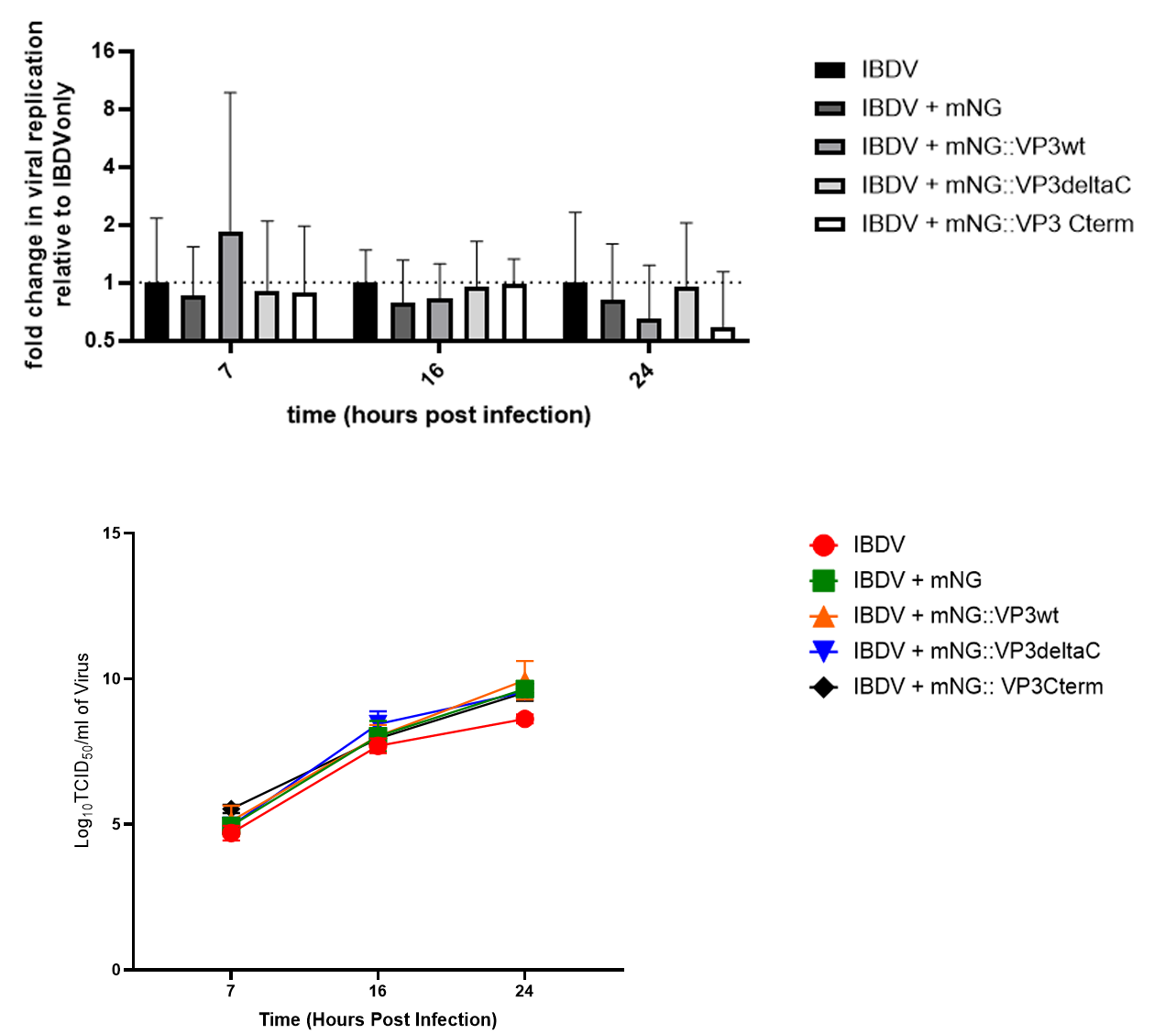


**Fig. S4. Exogenous expression of VP3 and VP3 variants do not significantly impact IBDV replication kinetics.** DF-1 cells were infected with IBDV strain PBG98 alone or infected with IBDV strain PBG98 and simultaneously transfected with plasmids expressing mNG, mNG::VP3, mNG::VP3$\Delta$C or mNG::Cterm in triplicate. Cells were lyse, RNA extracted at 7, 16, and 24hpi, and viral replication was quantified by RTqPCR and plotted as fold change normalized to IBDV infection alone (A). The titer of virus in the supernatant from the same samples was also assessed by TCID_50_, and the resultant growth curve plotted (B).


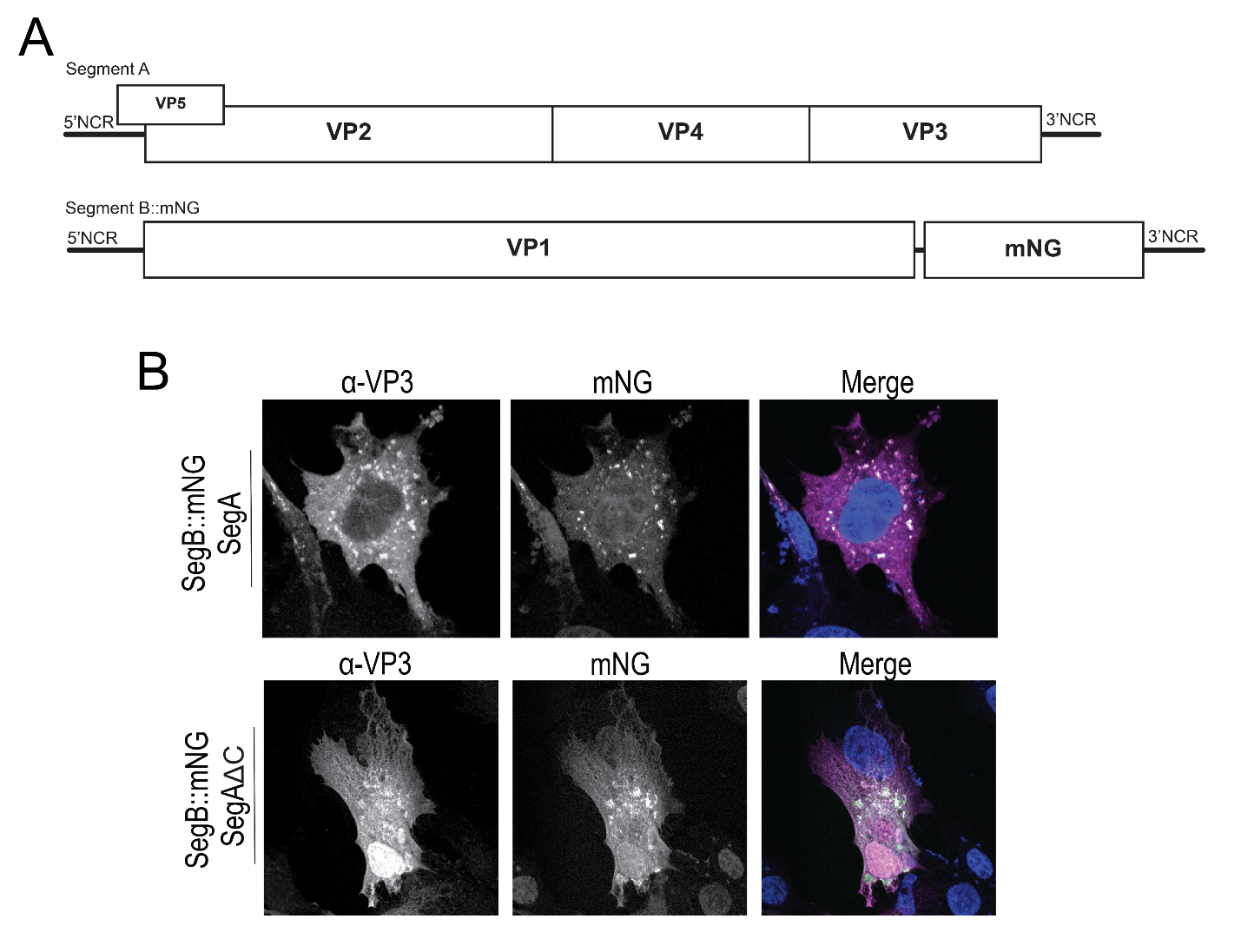


**Fig. S5. A schematic of the reporter tagged system that permitted live cell imaging.** pIBDV-SegA displayed on the top and pIBDV-SegB::mNG on the bottom (A). DF-1 cells were co-transfected with a mixture of pIBDV-SegB::mNG and either pIBDV-SegA or pIBDV-SegA$\Delta$C, fixed at 34hpt, and immunostained with anti- VP3 (nuclei stained with DAPI). The signal from individual channels shown in black and white, with the merged image in color (B).


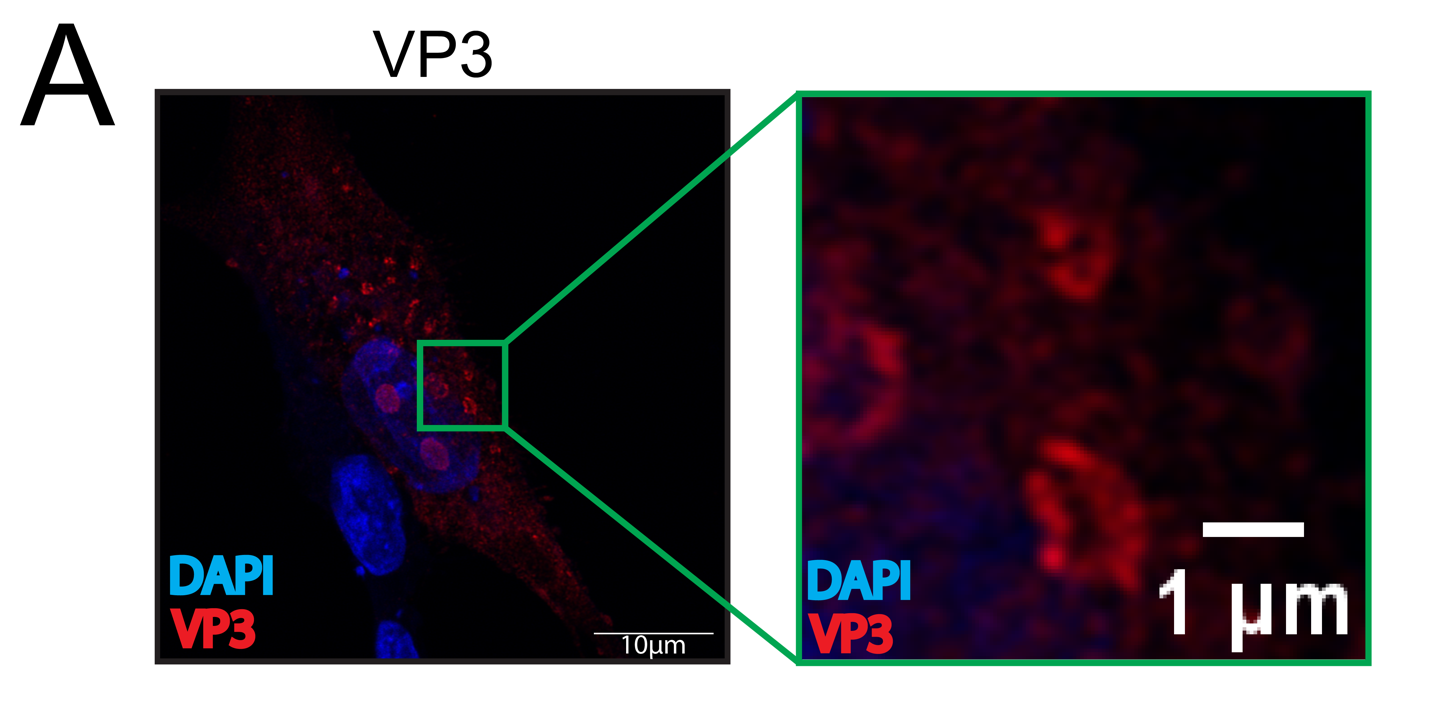


**Fig. S6. A subset of VP3-expressing cells display annular puncta.** DF-1 cells were transfected with SegA alone, stained with a monoclonal antibody against VP3, and imaged with a Zeiss LSM980 Airyscan 2 super resolution confocal microscope. A subset of VP3-positive cells exhibited an “annular” phenotype.


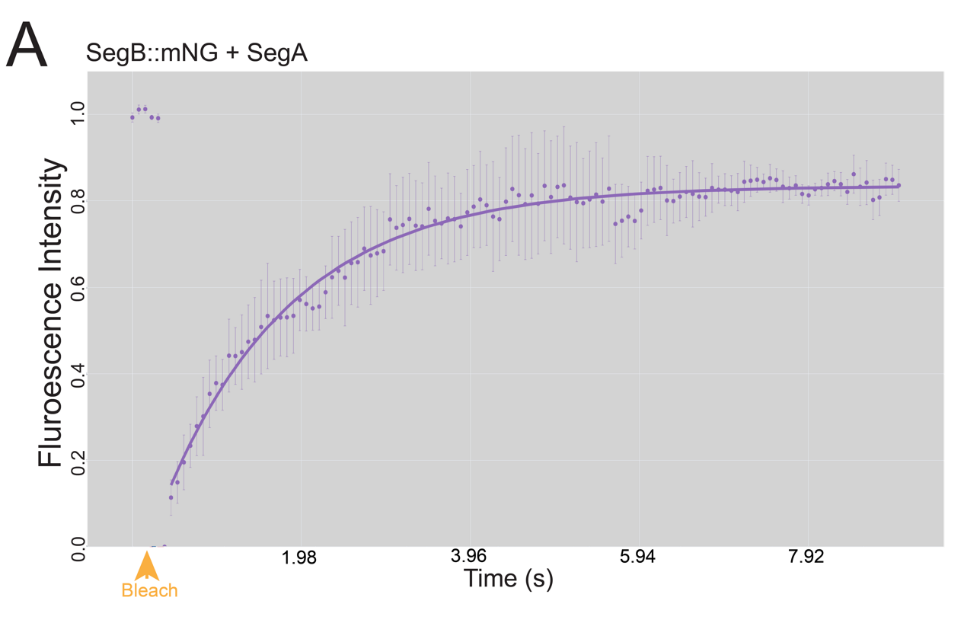


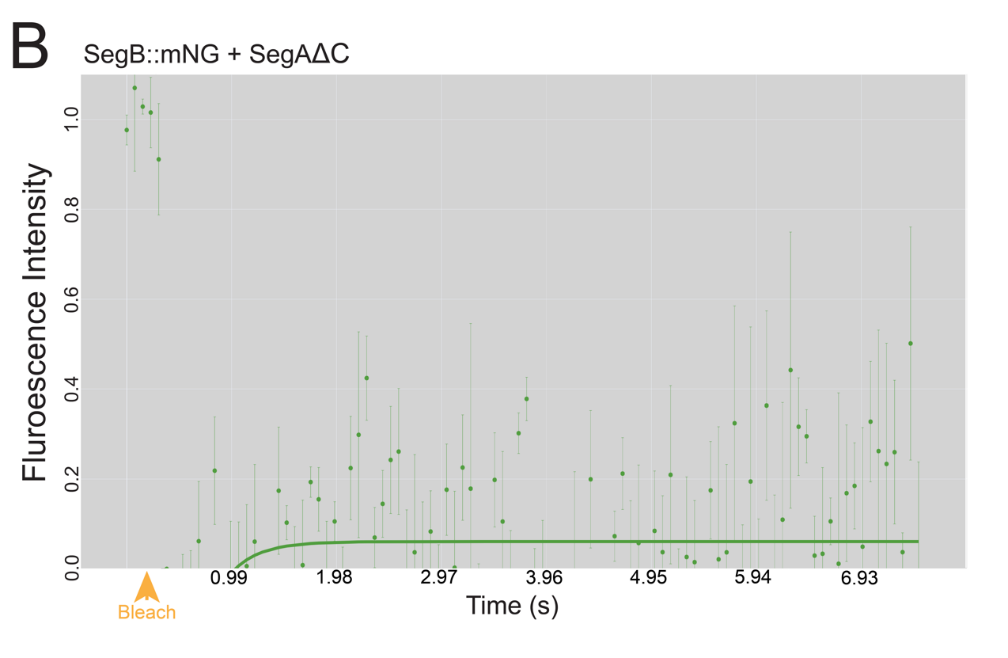


**Fig. S7.** **The VP3 C terminal IDR significantly contributes to the mobile fraction of the molecules within the cytoplasmic puncta in a macrophage cell line (HD11).** HD11 cells were co- transfected with a mixture of SegA and SegB::mNG, and a time lapse series was captured by live confocal microscopy at 34 hpt with a single iteration of point bleaching on the 5^th^ frame. Intensity data was extracted and used to calculate a FRAP recovery curve. Results are from 3 replicates; error bars = SEM (A). HD11 cells were co- transfected with a mixture of SegA$\Delta$C and SegB::mNG and a FRAP recovery curve generated in the same way (B).

**Movie S1.** Animated 3D scatter plot representing the positions of the alpha carbons across a single 20 nanosecond molecular dynamics simulation of the Alfafold2-derived structure of the VP3 monomer. Each frame represents 10 picoseconds of elapsed time. The colors of the points are representative of atomic velocity, with blue being the minimal observed velocity, and red being the maximum observed velocity.

**Movie S2**. Animated 3D scatter plot representing the positions of alpha carbons across a single 20 nanosecond molecular dynamics simulation of the Alphafold3-derived structure of the VP3 homodimer. Each frame represents 10 picoseconds of elapsed time. The colors of the points are representative of atomic velocity, with blue being the minimal observed velocity, and red being the maximum observed velocity.

**Supplemental Materials and Methods.**

**Sequence Entropy.** Briefly, sequence entropy was calculated using a sliding-window approach, where for each residue at position $i$ in a sequence of length $n$, the bounds of a window with size $w$ are defined as:

$$W_{i}= \left[ \max\left( 0, i-\frac{w}{2} \right),\min\left( n, i+ \frac{w}{2}+1 \right) \right]$$

This formulation allowed for the window size $w$ to be set to 20 residues and be resized dynamically when a 20 residue window centered on $i$ was not possible due to the position of $i$. For each residue $a$ within $W_{i}$, the probability $p(a)$ was given by the equation:

$$p\left( a \right)= \frac{F(a)}{\mathrm{length}(W_{i})}$$

Where $F(a)$ is the frequency of residue $a$. The Shannon entropy $H$ at each position $i$ was then calculated from the values of $p(a)$ by the equation:

$$H_{i}= -\sum_{a\in W_{i}} p\left( a \right)\log_{2} p\left( a \right)$$

These values were then normalized to give an entropy score $E$ by the equation:

$$E_{i}= \frac{H_{i}}{max(H)}$$

**Molecular Dynamics (MD) simulations: Periodic Boundary Conditions (PBC).** As PBC were employed in MD simulations, the absolute positions of atoms in trajectories were corrected by an algorithm. This algorithm ensured that in a simulation box $d$ with dimensions $d_{x}, d_{y}, d_{z}$ , for each atom $i$ of $M$ total atoms, at each time step $t$ of $N$ total time steps, no translation of $r_{i}\left( t+1 \right)$ in $x$, $y$, or $z$ by an integer multiple of $d_{x}$, $d_{y}$, or $d_{z}$ respectively results in a position ${r_{i}}^{'} \left( t+1 \right)$ where

$\left| {r_{i}}^{'} \left( t+1 \right)- r_{i}(t) \right|<\left| r_{i} \left( t+1 \right)- r_{i}(t) \right|$. If such a translation is found, it is applied. The coordinate space for each frame was then normalized by uniformly translating the positions $r$ such that the centroid of all points on each frame lay at $(\frac{d_{x}}{2}, \frac{d_{y}}{2}, \frac{d_{z}}{2})$.

**MD simulations: B factor calculations.** The B factor was calculated from the MD trajectories by determining the mean position of each alpha-carbon across all frames of the simulation, given by the equation:

$$\left\langle r_{i} \right\rangle= \frac{1}{N}\sum_{t=1}^{N} r_{i}(t)$$

Where N is the total number of time steps for the simulation, and $r_{i}(t)$ is the position of alpha carbon $r_{i}$ at time $t$. The mean squared displacement (MSD) was then calculated for the simulation on a per-atom basis, given by the equation:

$$\left\langle\left( r_{i}- \left\langle r_{i} \right\rangle\right)^{2} \right\rangle= \frac{1}{N}\sum_{t=1}^{N} \left( r_{i}(t)- \left\langle r_{i} \right\rangle\right)^{2}$$

This MSD value was then converted to a B factor value for the $i^{th}$ atom by the equation:

$$B_{i}= \frac{8\pi^{2}\left\langle\left( r_{i}- \left\langle r_{i} \right\rangle\right)^{2} \right\rangle}{3}$$

To perform residue:residue statistical analysis of the B factor values, the B factor value $B_{i}$ for the alpha carbon of each residue $i$ was calculated as described for each of 6 independent simulations. For each residue, the B factor values were tested for normality by a Shapiro-Wilk test, with a significance threshold $\alpha$ of 0.05. As not all values $p_{i}$ met the significance threshold, the non-parametric Kruskal-Wallis test was performed across the groups, with a significance threshold $\alpha$ of 0.05. Given that $p_{kruskal} \ll0.001$, *post-hoc* pairwise testing was performed using Dunn’s test, with Benjamini-Hochberg correction.

**MD simulations: Atomic motion correlation analysis.** The pairwise Pearson’s product-moment correlation coefficients ($r_{xy}$) were calculated for the fluctuations of all alpha carbons about their mean positions for each MD simulation. For statistical comparison, the mean absolute value of the correlation coefficients for each pair of alpha carbons (excluding self-comparisons) in the C-terminus was compared to that of an equally-sized (36 residue) range centered on each residue of the structure where such a window was possible. A Shapiro-Wilk test was performed on each range and the null hypothesis strictly failed to be rejected at a significance level $\alpha=0.05$, and Levene’s test was performed between each range and the C-terminus, however the null hypothesis was rejected for some ranges. As an assumption of normality but not of homoscedasticity was supported, mean correlation coefficients were compared by a one-tailed Welch’s unequal variances *t-*test with the alternate hypothesis $H_{a}: \bar{\left| r_{ab} \right|}>\bar{\left| r_{cd} \right|} a,b\in{range}_{test} and c, d\in{range}_{cterm}.$ The null hypothesis was rejected at a significance level $\alpha=0.05$ for all ranges except those centered on residues 74 – 96 and 149-163, both of which correspond to predicted unstructured regions of the protein, and the latter of which was previously found to be unstructured by X-ray crystallography. For completeness, this analysis was also performed using a dynamically sized test window to permit analysis of windows centered on residues at the N and C extrema. Although statistical tests should be considered less reliable as the window, and therefore sample size decreases, the conclusions of the analysis were unchanged.

**FRAP.** Briefly, background fluorescence intensity was compensated for in the ROI and whole puncta by subtracting it at each time point $t$, as given by the equations:

$${{I(t)}_{ROI}}^{'}= {I(t)}_{ROI}- {I(t)}_{BG}$$

$${{I(t)}_{VF}}^{'}= {I(t)}_{VF}- {I(t)}_{BG}$$

The “double normalized” fluorescence intensity was then calculated using the equation:

$${I(t)}^{double}= \left( \frac{\frac{1}{n_{pre}}\cdot\sum_{t=1}^{n_{pre}} {{I(t)}_{VF}}^{'}}{{{I(t)}_{VF}}^{'}} \right)\cdot\left( \frac{{{I(t)}_{ROI}}^{'}}{\frac{1}{n_{pre}}\cdot\sum_{t=1}^{n_{pre}} {{I(t)}_{ROI}}^{'}} \right)$$

Where $n_{pre}$ is the number of pre-bleach frames. The “full-scale normalized” intensity was then calculated using the equation:

$${I(t)}^{fullscale}= \frac{{I(t)}^{double}-{I(t_{postbleach})}^{double}}{1- {I(t_{postbleach})}^{double}}$$

Where $t_{postbleach}$ is the first post-bleach timepoint. Curve parameters were then determined from this data by least-squares curve fitting, with the equations:

$$I_{single}= I_{0}-a\cdot e^{-\beta t}$$

$$I_{double}= I_{0}-a\cdot e^{-\beta t}- \gamma\cdot e^{-\delta t}$$

Which represent single and double term exponential curves respectively. As part of least-squares curve fitting, initial values of $I_{0}=0.85$, $\alpha=0.5$, $\beta=0.563$, $\gamma=0.316$, and $\delta=0.36$ were used. As full-scale normalization was employed, mobile fraction $mf$ was determined directly from the curve parameters, as $mf= I_{0}$for full-scale normalized data. The half-maximal recovery time $t_{half}$ or $t_{\frac{1}{2}}$ for a single-exponential fit is given by the equation:

$$t_{half}=\frac{\ln2}{\beta}$$

Whereas for double-exponential fits, $t_{half}$ is computed numerically. Choice of exponential equation was made by comparing coefficients of determination ($R^{2}$), as determined by the equation:

$$R^{2}=1- \frac{\sum_{t}^{n} \left( {I(t)}^{fullscale}-f(t) \right)^{2}}{\sum_{t}^{n} \left( {{I(t)}^{fullscale}- \left( \frac{1}{n}\sum_{t}^{n} f(t) \right)}^{2} \right)}$$

Where $f(t)$ is the value of the chosen exponential equation $f$ with the calculated curve parameters at time $t$.
